# Factors Defining Human Adipose Stem/Stromal Cell Immunomodulation in Vitro

**DOI:** 10.1007/s12015-023-10654-7

**Published:** 2023-11-14

**Authors:** Marwa Mahmoud, Mazen Abdel-Rasheed, Eman Reda Galal, Rehab R. El-Awady

**Affiliations:** 1 Stem Cell Research Group, Medical Research Centre of Excellence, National Research Centre, 33 El Buhouth St, Ad Doqi, Dokki, 12622 Cairo Governorate Egypt; 2 Department of Medical Molecular Genetics, Human Genetics and Genome Research Institute, National Research Centre, Cairo, Egypt; 3 Department of Reproductive Health Research, National Research Centre, Cairo, Egypt; 4 Department of Biochemistry and Molecular Biology, Faculty of Pharmacy (Girls), Al-Azhar University, Cairo, Egypt

**Keywords:** Human adipose stem/stromal cells, Immune cells, Coculture, Immunomodulation, Preconditioning, Experimental determinants

## Abstract

Human adipose tissue-derived stem/stromal cells (hASCs) are adult multipotent mesenchymal stem/stromal cells with immunomodulatory capacities. Here, we present up-to-date knowledge on the impact of different experimental and donor-related factors on hASC immunoregulatory functions in vitro. The experimental determinants include the immunological status of hASCs relative to target immune cells, contact vs. contactless interaction, and oxygen tension. Factors such as the ratio of hASCs to immune cells, the cellular context, the immune cell activation status, and coculture duration are also discussed. Conditioning of hASCs with different approaches before interaction with immune cells, hASC culture in xenogenic or xenofree culture medium, hASC culture in two-dimension vs. three-dimension with biomaterials, and the hASC passage number are among the experimental parameters that greatly may impact the hASC immunosuppressive potential in vitro, thus, they are also considered. Moreover, the influence of donor-related characteristics such as age, sex, and health status on hASC immunomodulation in vitro is reviewed. By analysis of the literature studies, most of the indicated determinants have been investigated in broad non-standardized ranges, so the results are not univocal. Clear conclusions cannot be drawn for the fine-tuned scenarios of many important factors to set a standard hASC immunopotency assay. Such variability needs to be carefully considered in further standardized research. Importantly, field experts’ opinions may help to make it clearer.

**Graphical Abstract:**

Parameters that promote ASC immunosuppression on immune cells. Activation of immune cells induces their proliferation and differentiation and presence of ASCs modulates/suppresses such consequences. Augmented immunosuppressive effects of ASCs can be introduced in direct contact with the immune cells and via complementing the repeatedly reported experimental settings (texts in grey shapes). Abbreviations: ASCs: adipose tissue-derived stem/stromal cells, IFN-ɤ: Interferon gamma, MLR: Mixed lymphocyte reaction, TNF: Tumor necrosis factor.

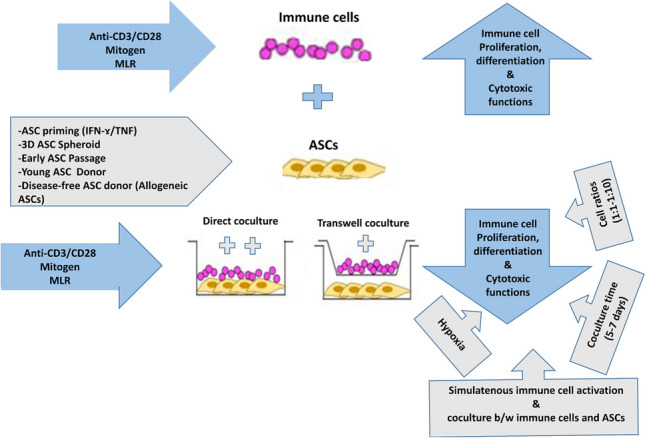

**Supplementary Information:**

The online version contains supplementary material available at 10.1007/s12015-023-10654-7.

## Introduction

Adipose tissue-derived stem/stromal cells (ASCs) are the relatively homogenous population of fibroblast-like cells that can be expanded after plating the stromal vascular fraction of adipose tissue (AT) onto standard cell culture surfaces [1]. hASCs express the surface markers of mesenchymal stem/stromal cells (MSCs), including the receptor molecules CD90 and CD105; the glycosyl phosphatidylinositol-anchored enzyme CD73 and the cell adhesion molecules CD29, CD44, CD146, and CD166. Additionally, hASCs should be negative for the hematopoietic antigens, including CD11b, CD13, CD14, CD19, and CD45, the endothelial markers CD31 and CD34, and the human leukocyte antigen (HLA)-DR [2].

**Fig. 1:**
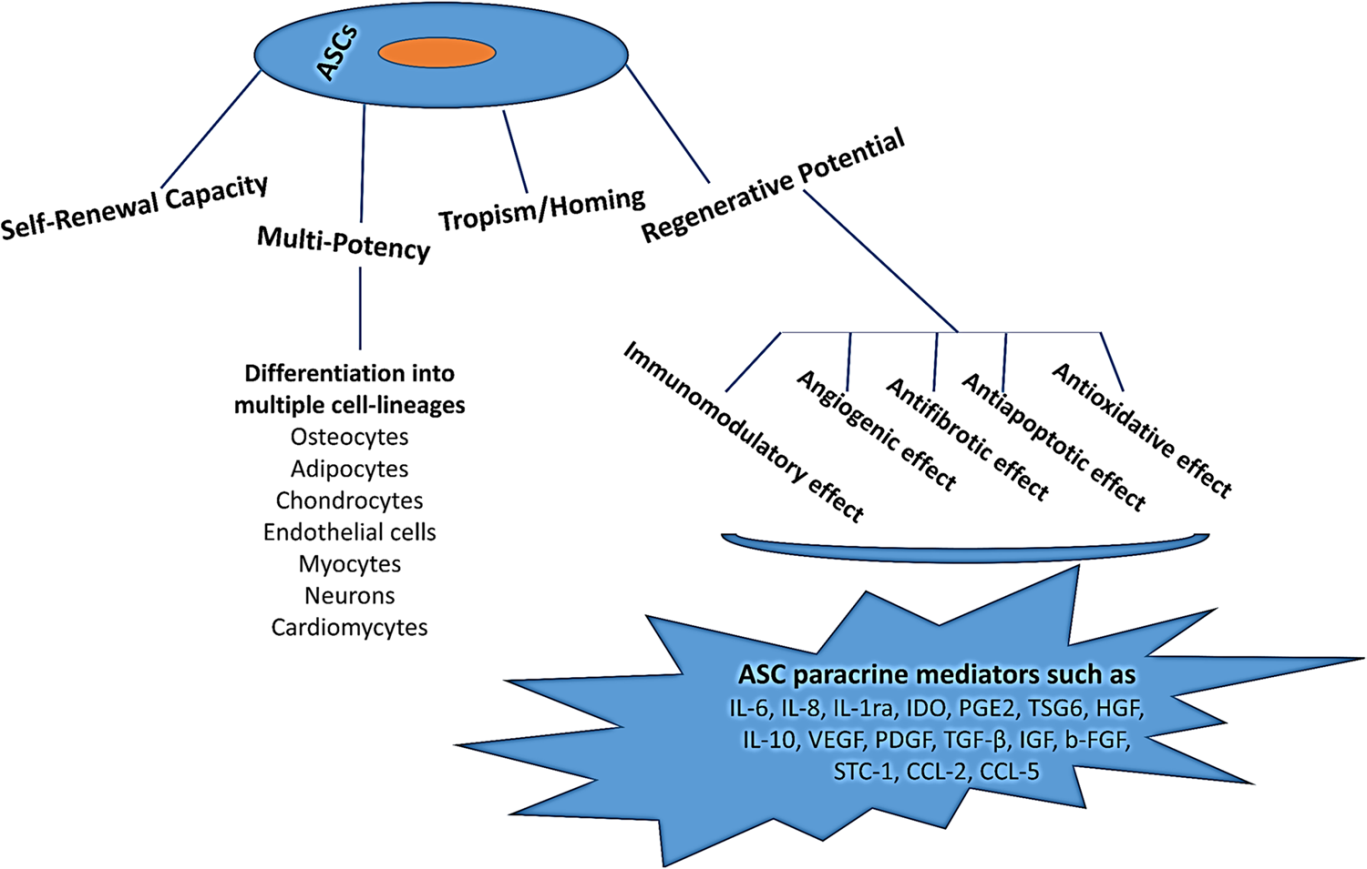
ASCs’ regenerative characteristics*.* Abbreviations: ASCs: adipose tissue-derived stem/stromal cells, b-FGF: basic Fibroblast growth factor, CCL2: C–C motif chemokine ligand 2, CCL5:: C–C motif chemokine ligand 5, HGF: Hepatocyte growth factor, IL-6: Interleukin 6, IL-8: Interleukin 8, IL-1ra: Interleukin 1 receptor antagonist, IL-10: Interleukin 10, IGF: Insulin-like growth factor, IDO: Indoleamine 2, 3 dioxygenase, PGE2: Prostaglandin E2, PDGF: Platelet-derived growth factor, STC-1, stanniocalcin-1, TSG-6: Tumor necrosis factor stimulated gene-6, TGF-β: Transforming growth factor beta, VEGF: Vascular endothelial growth factor

ASCs hold great promise for clinical application as a personalized cell therapy because of a number of advantageous characteristics (Fig. 1).They can be easily isolated with minimal ethical issues and donor risk and they are expandable in vitro [3, 4]. hASCs exhibit more enhanced proliferation, multipotency [5], and immunosuppressive capacity [6], however, lower senescence [7], than donor-matched bone marrow (BM)-derived MSCs (BMSCs). Importantly, varied paracrine factors including inflammatory, angiogenic, anti-apoptotic, anti-oxidative, anti-fibrotic, and anti-inflammatory mediators, contribute to ASCs-mediated tissue repair [8, 9].

ASCs are widely reported not to induce the immune response of allogeneic lymphocytes [10–12]. Such property was attributed to the low expression of major histocompatibility (MHC) class II molecule (MHC II/HLA-DR) and co-stimulatory molecules, CD40, CD80, and CD86 [12, 13]. On the contrary, some studies illustrated that ASCs had the potential to activate the proliferation of resting allogeneic CD4 T cells under circumstances of low inflammation [14, 15], and to induce the reactivity of cytotoxic CD8 T cells educated with allogeneic ASCs [16], or the production of alloreactive-memory CD8 T cells [17], so they are not intrinsically immunoprevilliged [18].

The immunomodulatory functions of hASCs in vitro are multifaceted and include the proliferation and differentiation of a variety of immune cells [19]. In particular, the effect of hASCs on effector T helper (Th) cells and regulatory T cells (Tregs) has been widely studied [20–22] (Supplemental Table 1). ASCs inhibit the proliferation of T cells via a plethora of paracrine mechanisms, including indoleamine 2, 3-dioxygenase 1 (IDO) activity [22, 23], secretion of prostaglandin E2 (PGE2) [24], leukemia inhibitory factor (LIF) [25], tumor necrosis factor-stimulated gene 6 (TSG-6) [26], interleukin 1-receptor antagonist (IL-1RA) [27], and other several factors [19, 28], and also induce T cells to adopt a regulatory phenotype [29, 30]. Surface molecules also contribute to ASC immunosuppressive effect on T lymphocytes [31–33].

In addition, hASCs have been reported to affect the proliferation, differentiation, and immune functions of B cells [34], inhibit dendritic cell (DC) maturation [35], suppress natural killer (NK) cells cytotoxicity [36, 37], and stimulate macrophage polarization to anti-inflammatory macrophages [38, 39]. It has been recently evolved that apoptotic MSCs, after MSC infusion, are phagocytosed by macrophages that are then reprogrammed to become immunoregulatory cells [40].

**Fig. 2:**
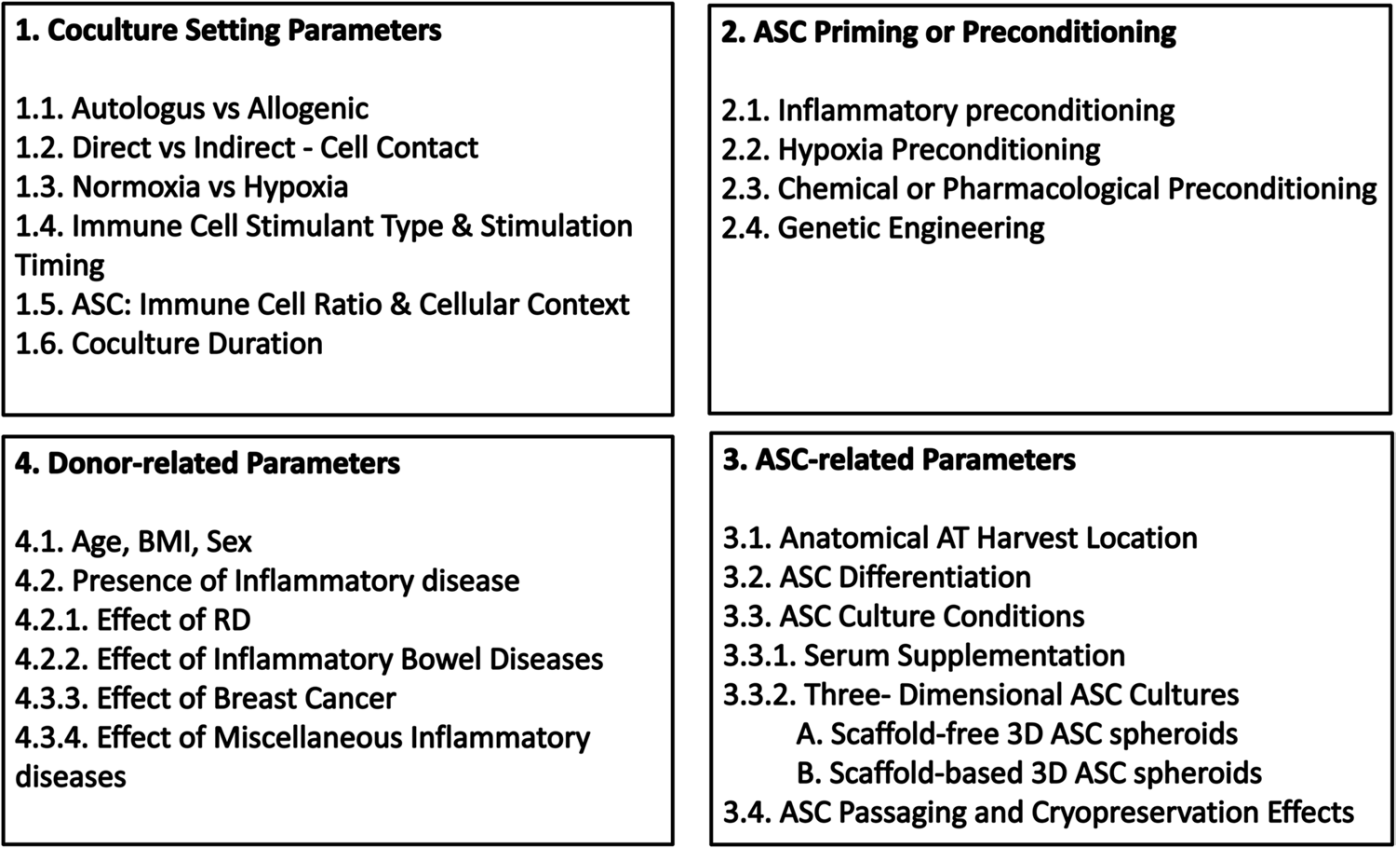
Factors affecting hASC immunomodulation in vitro*.* Abbreviations: hASCs: human adipose tissue-derived stem/stromal cells, AT: adipose tissue, 2D: two-dimensional, 3D: three-dimensional, BMI: body mass index

The above findings recommend the potential immunomodulatory ability of ASCs in vitro [41, 42]. However, the ASC immunomodulation in culture greatly depend on multiple parameters. In the current review, the impact of the experimental settings and donor characteristics on the immunomodulatory effects of hASCs in vitro are discussed. Factors that may control hASC immunomodulatory phenotype and functions in vitro are categorized into four main groups including coculture setting parameters, ASC priming or preconditioning, ASC-related parameters, and donor-related characteristics (Fig. 2).

## Coculture Setting Parameters

### Autologous vs. Allogeneic

Despite the off-shelf availability of allogeneic MSCs, patient-derived (autologous) rather than allogeneic MSCs may be the safer choice in clinical perspectives, to avoid anti-donor immune responses in some cases [43, 44]. Human ASCs are able to modulate the activity of both autologous and allogeneic immune cells in vitro; however, the strength of the suppressive effect may be different [45]. ASCs isolated from patients with rheumatic diseases (RD) were able to inhibit the proliferation [46], and to attenuate the expression of the activation marker CD25 on allogeneic phytohemagglutinin (PHA)-activated CD4 and CD8 T lymphocytes [47]. Relative to that setting, the RD ASCs comparably inhibited the proliferation [46], however, a weaker inhibitory effect to downmodulate CD25 expression on autologous PHA-CD4 and CD8 T lymphocytes [47]. Such results suggest a possible weaker control of T cell activation by autologous ASCs of RD patients in vivo [47]. In the context of diabetes mellitus, hASCs, from patients with type 2 diabetes (T2D), exhibited compromised antiproliferative potential on autologous anti-CD3/CD28-activated peripheral blood mononuclear cells (PBMCs) [48]. However, co-transplantation of autologous ASC-derived insulin-producing cells and hematopoietic stem cells showed a better response in individuals with type 1 diabetes (T1D) as compared with a similar allogeneic regimen [49]. Recently, it has been assumed that the current clinical scenario recommend the suitability of using autologous MSCs for T1D, not T2D therapy [50]. We think that numerous standardized studies to address the in vitro immunomodulatory phenotypes and functions of hASCs, from patients with different inflammatory diseases in autologous settings, are still needed, before assumption of the possibility of using autologous ASCs therapy in immune-related or inflammatory diseases.

### Direct- vs. Indirect- Cell Contact

Some studies revealed the necessity of direct contact between (b/w) hASCs and immune cells to exert their immunoregulatory effect [51–54], where transwell experiments decreased the strength of inhibition of hASCs on mitogen-activated PBMCs [51, 55], not on a mixed lymphocyte reaction (MLR) [51]. Quaedackers et al. studied the impact of cell-to-cell interactions b/w ASCs and activated T cells on the immunomodulatory potential of the former cell population [56]. The authors reported that allogeneic activation of PBMCs had induced the attachment of their membranes to ASCs after 1h and 24h of coculture. The cell binding was HLA-class I or class II independent, as similar interactions had been established b/w the PBMCs and autologous or allogenic ASCs, without the additional effect of ASC treatment with the pro-inflammatory cytokine; interferon-gamma (IFN-ɤ). Analysis of ASC-bound and suspended lymphocytes revealed that the former lymphocytes had been enriched for B cells, CD8^+^ T cells, and CD4^−^ CD8^−^ T cells, whereas CD4^+^ T cell-bound proportion had been increasing over time. The bound CD4^+^ T cells were well-proliferative, and highly activated, in terms of CD25 elevated expression, they also expressed the regulatory transcription factor, forkhead box P3 (FOXP3), and however, expression of CD127 excluded the Treg phenotype (CD4^+^ CD25^high^ CD127^low^ FOXP3^+^) [57]. Cell proximity potentiated the immunosuppressive effect of the cell membrane or soluble ASC immunomodulatory proteins on IL-2-treated CD8^+^ T cells. ASCs depressed the bound and suspended CD8^+^ T cells' response to IL-2, as presented by a reduced increase in the level of phosphorylated signal transducer and activator of transcription (STAT)-5, with a more pronounced effect in the bound cells. The results indicated that in an active immune environment, ASCs secrete attractant and bind T lymphocytes inhibiting CD8^+^ T cell activity and depleting the activated CD4^+^T cells from the cell suspension compartment [56]. In another study, direct contact b/w activated CD3^+^ T cells and hASCs led to active attachment and flattening of T cells to ASCs surfaces, specifically CD3^+^/CD4^+^ Th cells and NK T cells (CD3^+^/CD56^+^16^+^). At the attachment sites, elevated expression of intracellular adhesion molecule 1 (ICAM-1)/CD54 was detected, indicating the formation of highly specific ligand-receptor contacts b/w interacting cells [58]. Recently, ASC spheroids modulated effectively the proliferation and cytokine production of allogenic γδ CD3^+^ T lymphocytes in direct contact, while their conditioned medium (CM) induced much weaker inhibitory effects [59].

Other reports demonstrated that direct interaction is not essential for the hASC immunosuppressive effects, as the immunoinhibition persisted in transwell experiments where hASCs and immune cells were separated by a semipermeable membrane [22, 23, 32, 58, 60–66]. CM from IFN-ɤ treated hASCs were able to abrogate the proliferation of anti-CD3/CD28 activated CD4 T cells [67]. Such studies confirm the contribution of paracrine soluble factors and extracellular vesicles to hASC immunosuppressive effects [19].

### Normoxia vs. Hypoxia

A number of investigators addressed the immunoregulatory impact of hASCs under hypoxic (1–7%) vs. normoxic or ambient oxygen (O2) levels (20%) [58, 68–73]. Low O2 level is typical for the putative site of the MSC-T cell interactions [72]. Hypoxia potentiated the direct antiproliferative effect of hASCs on PHA-activated PBMCs [70] or CD3^+^ T cells [58, 71]. The effective hASC antiproliferative potential at 5% over 20% O2 level was attributed to the upregulated expression of *PDCD1* and *TGF-β1*, in cocultured T cells, which encode for programmed cell death protein 1 (PD-1) and TGF-β1, respectively [58], and both markers are involved in cell cycle arrest [74, 75]. Moreover, hASCs superior suppression for IFN-ɤ secretion by the directly cocultured T cells at low O2 level [58]. Conclusively, cell-to-cell contact may induce stronger hASC immunosuppression at 1% or 5% O2 over 20% O2 level [58, 70].

### Immune Cell Stimulant Type & Stimulation Timing

The MSC populations act as a switcher of inflammation, at a low level of immune cell activation, they acquire a pro-inflammatory phenotype, while at a high level of inflammation, they are immunosuppresors [76]. Similarly, ASCs are plastic immunoregulators that depend on the stimuli context to acquire pro- or anti-inflammatory phenotype [77]. Thus, the hASC immunoregulatory functions in vitro are affected by the type of immune cell stimulant (mitogen vs. alloantigen/MLR) [51, 61]. ASC immunosuppression on PBMCs was the greatest against proliferation induced by PHA, then by concanavalin A, and last by OKT3 (anti-CD3 antibody) [51].

Another determinant is the level of immune cell activation which is defined by the timing of coculture relative to stimulation, i.e. simultaneous coculture and stimulation vs. coculture after stimulation induction [78]. Mancheño-Corvo et al. [78], investigated the influence of PBMC pre-stimulation on the antiproliferative effect of hASCs. T lymphocyte pre-stimulation impaired in a time-dependent manner the capacity of ASCs to inhibit proliferation. Deficient ASC potential to inhibit the proliferation of 48 h pre-stimulated PBMCs was not due to reduced *IDO* activity, but rather to the kinetics of tryptophan (Trp) degradation and the low level of Trp available in the medium at 48 h of stimulation to be degraded by IDO. Pre-activation of ASCs with IFN-ɤ or polyinocinic-polycytidykic acid (poly I:C), toll-like receptor (TLR)-3 ligand, restored their capacity to inhibit proliferation of 48 h pre-stimulated lymphocytes, with a stronger effect with IFN-ɤ [78].

### ASC: Immune Cell Ratio & Cellular Context

The immunomodulatory potential of hASCs in vitro is, as well, strongly affected by cells ratio. At a hASC: immune cell ratio ranging from (1:1–1:25), hASCs exhibit potent immunosuppressive properties on diverse immune cell types. Substantial literature studies reported maximum hASC immunosuppression at high ASC: immune cell ratio (from 1:1 to 1:10) [6, 10–12, 14, 21, 24, 25, 33, 36, 51, 58, 61, 64, 67, 73, 79–88]. Others reported maximum suppression at lower ratios (1:20) [89] or (1:25) [23, 32, 35, 90]. At cell ratios 1:50, 1:100, or 1:1000, hASCs inhibited [6, 51] or failed to inhibit the proliferation [14, 23] of activated lymphocytes. The attenuated hASC immunosuppression at low ASC densities could be associated with intensive cell death [91], and autophagy-mediated apoptosis of MSC under inflammatory conditions [92]. Surprisingly, Th17 lymphocyte pathway is significantly modulated by hASC density, and it was greatly enhanced at high (1:5), compared with low (1:80) ASC: T cell ratio, contradicting the well‐documented immunosuppressive effect of ASCs, specifically at high density [93].

The intensity of ASCs inhibition varied with cellular contexts, i.e. the whole PBMC population or a specific purified immune cell compartment is included in the coculture with the hASCs [22, 45, 61]. The effect of the cellular context on the in vitro ASC immunoregulation was clearly presented in cocultures of hASCs and mitogen (PHA)-stimulated PBMCs, where there was a marked decrease of IFN-ɤ and a significant increase of IL-17AF. While, in the cocultures of anti-CD3/CD28 activated CD4 + T cells with ASCs, there was some increase in IFN-ɤ and IL-17AF. As well, ASCs significantly downregulated CD25 expression on PHA-stimulated PBMCs, however, they did not affect its expression on ɑ-CD3/CD28-activated T cells [61]. Such findings recommend that immune cells create a cytokine milieu in vitro which differs depending on the stimulation method and cellular context, and in turn differentially affects ASC immunomodulatory actions [61]. To shift Th differentiation to a functional anti-inflammatory direction, ASCs require accessory cell support, whereas their direct effect on a purified immune cell type may be a pro-inflammatory [46]. In other contradictory reports, hASCs exerted analogous immunosuppressive action on the proliferation of activated whole PBMCs population and enriched CD4^+^ T cells [22, 67], reducing the impact of the interplay of accessory immune cells (B cells, NK cells, and monocytes) within PBMCs on MSC immunoregulation [94].

### Coculture Duration (Assessment Day)

The duration of the ASCs/immune cells interaction is another considerable issue. The type of immune cells [64] and the assessed immune cell-related parameters [33], are determinants of the coculture time. Significant inhibitory effects of hASCs have been reported on days 3 [36, 58, 70], 4 [33, 95], 5 [6, 23–25, 46, 51, 96], 7 [21], or 10 [34], of coculture. Inhibition of lymphocytes’ immune responses becomes more distinct with an extension of the coculture time [45, 51].

## ASC Priming/Licensing/Preconditioning

MSCs can actively sense their surroundings and modulate, accordingly, their fate and behavior. Intriguingly, it has been proposed that MSCs immunosuppressive ability is not constitutive; instead, it is induced in inflammation [97, 98] and interaction with activated immune cells [99]. Several approaches have been introduced to boost the in vitro immunosuppressive functions of hASCs isolated from healthy donors or even patients with inflammatory diseases and they are discussed below.

### Inflammation Preconditioning

The cohabitation of ASCs with pro-inflammatory cytokines such as tumor necrosis factor (TNF)-α, IFN-ɤ, IL-6, IL-1β, and/or IL-17 can potentiate their effectiveness for inflammatory diseases [53, 100, 101]. Numerous investigators (Table 1) assessed the immunogenicity and/or immunosuppressive properties of hASCs after treatment with pro-inflammatory cytokine(s) [6, 13, 14, 22, 27, 61, 67, 85, 93, 102–121] or TLR agonist (s) [122–129].

**Table 1:** Studies that addressed the influence of inflammatory and/or hypoxia priming on the immunoregulatory phenotypes and functions of hASCs

Treatment conditions	Treatment period	ASCs’ response	The immunomodulatory functions of the primed ASCs	Reference
IFN-ɤ (50 ng/ml) + TNF-α (20 ng/ml) + IL-6 (10 ng/ml)	168 h	↑*IDO,CXCL-9, CXCL-10, CXCL-11*	−	[14]
TNF-α (10 ng/ml)	48 h	↑IL-6, IL-8, MCP-1 (CCL-2), CXCL-6, CXCL-2	Conditioned medium of TNF-α treated ASCs has also been reported to promote monocyte migration in vitro	[102]
IFN-ɤ (200 U/ml)	96 h	↑ PD-L1 (CD274), PD-L2, and ICAM-1 (CD54)	-Enhanced antiproliferative effect	[61]
IFN-ɤ (10^3^ U/ml) + TNF-α (50 ng/ml) + IL-1β (25ng/ml) + IFN-α (3 × 10^3^ U/ml)	Overnight	↑ PD-L1 (CD274)	‘/−	[103]
IFN-ɤ (500 U/ml)	4, 8, 24 h	↑ IDO	−	[6]
IFN-ɤ (10 ng/mL) TNF-α (15 ng/mL)	40 h	-↑ the immunosuppressive molecules [ *IDO*, *PTSG2* (Cox-2), *NOS2* (iNOS), and *TNFAIP6* (TSG-6) -↑ IDO activity	-Enhanced T and B-cell-suppressive capacities	[12]
IFN-ɤ (100 ng/ml)	24 h	↑ IDO expression and activity	-Enhanced antiproliferative effect on PBMCs, CD4 and CD8 T cells and enhanced reduction of IFN-ɤ, IL-6 and IL-1β production by stimulated PBMCs -Promoted immunosuppressive effect in a humanized skin graft model	[105]
IL-1β (20 ng/ml) + IL-6 (40 ng/ml) + IL-23 (20 ng/ml)	96 h	↑ TGF-β ↑ CD45	This priming strategy promoted CD45 expression by ASCs up to 80% and the CD45^+^ ASCs significantly abrogated the proliferation in MLR	[54]
IFN-ɤ (50 ng/ml) + TNF-α (50 ng/ml) + IL-1β (25ng/ml) + IFN-α (10 ng/ml)	Overnight	↑ *IDO,* and the immunoregulatory semaphorins *SEMA4D* and *SEMA7A*	−	[116]
IFN-ɤ + TNF-α (10, 20, or 40 ng/ml)	48 h	↑ IDO (dose-dependent increase) ↑ PGE2, IL-10 and IL-8 (20ng/ml) ↑ IL-6, CCL2 (40ng/ml)	-Exosomes from pre-treated ASCs with enhanced potential to revert M1 macrophages differentiation and to promote M2 macrophages polarization	[106]
IFN-ɤ (200 IU/mL)	24 h	↑ *IDO*	-Enhanced antiproliferative effect in vitro and immunoregulatory potential in the GVHD model	[85]
IFN-ɤ (10^3^ U/ml) + TNF-α (50 ng/ml) + IL-1β (25ng/ml) + IFN-α (3 × 10^3^ U/ml)	Overnight	↑ secretion of distinct Th17 related cytokines including IL-6, IL-1β, IL-17F, IL-21, and IL-23	-inflammatory priming and cell ratio significantly modulated the ASCs-mediated regulation of Th17 differentiation	[93]
Cytomix (TNF-α + IFN-ɤ + IL-1β, all at 5 ng/mL) + 21% O2 or 2% O2	48 h	↑ IL-1RA, IL-8, IL-6 and MCP-1 ↑*PTGS2*, *TNFAIP6*, and *STC-1*	At normoxia (21% O2), cytomix-treated ASCs induced more potent immunosuppressive functions than the relevant group at hypoxia	[27]
IFN-ɤ (50ng/ml)	48 h	↑ *IDO* and ICAM-1	-Enhanced antiproliferative effect of IFN-ɤ-ASCs and their CM -CM from INF-γ licensed ASCs had increased capacity to control the T-cell response	[108]
IFN-ɤ (50ng/ml) + TNF-α (50 ng/ml) + IL-1β (25ng/ml) + IFN-α (10ng/ml)	18 h	↑ HLA-ABC, CD40 CD54, CD106, CD274 and CD112	−	[13]
IFN-ɤ (10ng/ml)	72 h	↑ IDO expression and activity	-Priming enhanced the antiproliferative effects of hASCs on PBMCs and purified T cells	[22]
IFN-ɤ (100ng/ml)	48 h	↑ IDO, CD274 and IL-RA ↓ IL-1β	−	[109]
TNF-α (10 ng/ml) + IL-1β (10 ng/ml)	6 h followed by 48 h incubation in serum-free medium	↑IL-6, IL-8, MCP-1 (CCL2), CXCL6, CXCL2 and ECM proteins		[121]
IFN-ɤ (100ng/ml) + TNF-α (10 ng/ml)	24 h	↑ IDO, HLA-E, CD54 (ICAM-1), CD106 (VCAM-1), CD274 (PD-L1) and CD73 (NT5E)	−	[110]
TNF-α (50 ng/mL)	24 h vs 96 h	↑Genes related to immunomodulation (*TLR2* and *PTGS2*) and cell survival (*TRAF1, NF-kB,* and *IRF7*)	−	[111]
IL-1β (1ng/ml)	24 h	↑ Genes involved in inflammation modulation such as *CXCL6, CXCL8, LIF,* and *STC1* and extracellular matrix remodeling such as *MMP3, MMP10,* and* MMP1*	- IL-1β primed hASCs superiorly modulated the CD80^+^/CD206^+^ ratio in co-culture with polarized M1 proinflammatory macrophages, promoting an increase of the CD206^+^ M2a anti-inflammatory marker	[112]
IFN-ɤ (10ng/ml)	24 h	↑ IDO, CD274 and ICAM-1	The secretome of primed ASCs from health and T2D donors significantly suppressed the CD4 T cell proliferation	[67]
IFN-ɤ (3 ng/ml) or Poly I:C (10µg/ml)	48 h	↑ IDO activity	-Enhanced antiproliferative effect on 48 pre-stimulated T lymphocytes	[78]
TNF-α (10 ng/ml) or LPS (100 ng/ml)	24 h	↑IL-6, IL-8, and MCP-1	LPS-primed ASCs displayed enhanced therapeutic efficacy in skin flap survival in a diabetic rat model than did unprimed ASCs	[124]
LPS (10ng/ml) or Poly I: C (1μg/ml)	1 h	↑ IL-8	−	[126]
LPS (10ng/ml)	Up to 24 h	↑ TNF-α and GM-CSF in the secretome of the primed female line ↑ IL-6 in the secretome of the primed male line	The secretome of the female LPS-ASCs which contained a high level of GM-CSF promoted the CD14 monocyte differentiation in THP-1 cells	[127]
LPS (1μg/ml) or Poly I: C (10ng/μl)	ND	-TLR4 agonist ↑ *TLR4*, *IDO*, *TDO2*, *COX2*, *TGF-β1,* and *HGF* -TLR3 agonist ↑ *TLR3*, *IDO1*, *TDO2*, *COX2*, *TGF-β1,* and *HGF*	−	[128]
LPS (1μg/ml) or Poly I: C (10ng/μl)	1 h			[129]
5% O2 (Hypoxia)	72 h Followed by 16 h incubation in serum- free medium	↓ Type 2 cytokines (IL-13, MCP-1, and CD40 ligand)	Secretome with enhanced inflammation mitigation in fibrosis	[15]
IFN-ɤ (50 ng/mL) + TNF-α (20 ng/mL) + 1% O2	6, 24, 72 h	↑ IDO, CXCL10, PD-L1	-Enhanced inhibition of CD4^+^ and CD8^+^ T-cell proliferation	[71]
IFN-ɤ (100 ng/mL) + 1% O2	48 h	IFN-ɤ induced ↑ (IDO, PD-L1, HLA-E, HLA-G Hypoxia enhanced glucose consumption and production of T-cell inhibitory lactate levels	−	[130]

*ASC* adipose tissue-derived mesenchymal stem/stromal cells, *CD* Cluster of Differentiation, *CM* Conditioned medium, *COX/ PTSG* Cyclooxygenase/ Prostaglandin-endoperoxide synthase, *CXCL* chemokine (C-X-C motif) ligand, *ECM* Extracellular matrix, *GVHD* Graft versus host disease, *GM-CSF* Granulocyte macrophage-colony stimulating factor, *H* Hours, *HGF* Hepatocyte growth factor, *HLA* Human Lekocyte antigen, *hASCs* human ASCs, *IL* Interleukin, *IDO* Indoleamine 2,3 dioxygenase, *iNOS/NOS* Inducible nitric oxide synthase/ Nitric oxide synthase, *IFN-ɤ* Interferon-gamma, *IFN-α* Interferon-alpha, *ICAM* Intracellular cell adhesion molecule, *IRF* Interferon regulatory factor, *ILF* Leukemia inhibitory factor, *LPS* Lipopolysaccharide, *MCP* Monocyte chemotactic protein, *MMP* Matrix metalloproteinase, *NT5E* 5'-Nucleotidase, *NF-κB* nuclear factor kappa-light-chain-enhancer of activated B cells, *O2* oxygen, *PD-L* Programmed death ligand, *PGE2* Prostaglandin E2, *PBMCs* peripheral blood mononuclear cells, *poly I:C*Polyinosinic-Polycytidylic Acid, *STC* Stanniocalcin, *T2D* Type 2 diabetes, *TNF-ɑ* Tumor necrosis factor alpha, *TLR* Toll-like receptor, *TSG-6/TNFAIP6* TNF-stimulated gene 6/TNF-ɑ induced protein 6, *TGF-β* Transforming growth factor beta, *Th17* CD4 T helper 17 cells, *TRAF* TNF receptor-associated factor, *TDO2* Tryptophan-2, 3-dioxygenase 2, *VCAM* Vascular cell adhesion molecule

Pre-conditioning of ASCs with IFN-ɤ is one of the most common approaches to enhance ASC-mediated immunosuppression [19, 67, 105]. IFN-ɤ upregulates expression of the immunoregulatory surface molecules, programmed death-ligand 1 (PD-L1)/CD274, PD-L2, and ICAM-1/CD54 by hASCs [13, 61]. Moreover, IDO activity potentially involved in the enhanced immunosuppressive effects of IFN-ɤ-treated ASCs [14, 85]. Interestingly, ASCs showed a stronger upregulated expression of *IDO* than BMSCs by 8 h IFN-ɤ exposure [6]. Enhanced activation of the JAK/STAT1 pathway mediates IFN-ɤ induced expression of PD-L1 and IDO in primed MSCs [114]. From mechanistic perspectives, priming of MSCs with IFN-ɤ increased glucose turnover leading to abundant STAT1 glycosylation and stability, thus sustaining its downstream effects [115]. Glucose metabolic reprogramming is thus a novel modulatory mechanism for the immunosuppressive function of IFN-ɤ–challenged MSCs and this mechanism can be analyzed in the IFN-ɤ primed hASCs. Not only the IFN-ɤ licensed-ASCs, but also their CM can exert potential immunosuppression [67, 108]. Moreover, IFN-ɤ treatment using low [67] or high [109] dose enhanced the immunoregulatory phenotypes, and antiproliferative potential of ASCs from patients suffered from T2D with different ethnicities and body mass indices.

Individual treatment with TNF-α activated nuclear factor kappa B (NF-κB) pathway in hASCs to promote the cell survival. Transcriptome analysis revealed that TNF-α treated ASCs differentially expressed genes involved in the differentiation of ASCs into mononuclear leukocytes (*NFKB1, IRF8, RELA, RELB, IRF7*) and in the antitumor immunity (*TLR2* and *PTGS2)* [111]. Regarding treatment with IL-1β, priming of hASCs from patients with osteoarthritis (OA) with this cytokine (1 ng/ml for 24 h) induced the differential expression of genes enriched in inflammation modulation and extracellular matrix (ECM) remodeling. Moreover, compared to non-primed cells, IL-1β primed hASCs superiorly modulated the CD80^+^/CD206^+^ cell ratio in co-culture with polarized M1 macrophages, promoting an increase of the anti-inflammatory CD206^+^ M2a macrophages [112].

The synergistic effect of priming ASCs with a mixture of different inflammatory cytokines have been tested [13, 14, 27, 54, 93, 103, 106, 110, 116]. Full genome expression analysis was carried out for hASCs cultured for 7 days under control conditions and two different inflammatory conditions; either with alloactivated PBMCs (MLR) in a transwell setting or with a cocktail containing IFN-ɤ, TNF-α, and IL-6 [14]. Partial overlapping in the significant gene expression changes, induced by both inflammatory conditions, was demonstrated indicating different ASCs responses to alloactivated PBMCs than to pro-inflammatory cytokines. Human ASCs cocultured with MLR showed a significant upregulation of the PGE2-producing enzyme; cyclooxygenase 2 (*COX-2*) (tenfold). However, priming with the pro-inflammatory cytokines cocktail significantly induced the expression of *IDO* (394fold) in ASCs [14]. In another report, priming with a mix of IFN-ɤ, TNF-α, and IL-1β induced significantly the surface expression of CD274 and the secretion of PGE2 by hASCs [103]. The increased CD274 level under inflammatory conditions may be one of the mechanisms by which ASCs counteract the immunogenic effect of the upregulation of CD40 in inflammation [12, 103, 117]; these changes in expression may ultimately lead to inhibition of activated lymphocytes [103]. In addition to *IDO* upregulation, priming of hASCs with the cocktail (IFN-ɤ, TNF-α, IL-1β, and IFN-α) was found to upregulate the transcriptional levels of the immune regulatory semaphorins SEMA4D and SEMA7A [116]. The combination of IFN-ɤ/TNF-α induced significantly the release of PGE2, IL-10, and IL-8 by ASCs (at ≥ 20 ng/ml) and that of IL-6 and CCL-2, only at 40 ng/ml [106]. Cytomix treatment (IFN-ɤ, TNF-α, IL-1β, all at 5 ng/ml) at normoxia robustly induced the most potent immunosuppressive functions of ASCs, likely via induced release of IL-1RA, among other mediators [27].

The effect of human Th17 cell polarizing pro-inflammatory factors such as IL-1β, IL-6, and IL-23 on the immunophenotype and immunomodulatory properties of ASCs vs. BMSCs was studied [54]. It was found that priming of both MSC types with those factors promoted the expression of CD45 by about 80%. CD45^+^ ASCs and BMSCs maintained similarly the antiproliferative functions of the respective CD45^−^ MSCs on MLR, in contact- or contactless- dependent manner. Pro-inflammatory cytokines treatment did not modulate the secretion of IFN-ɤ, TNF-α by both MSC types, however, decreased that of IL-4 and increased that of TGF-β. Such increase in TGF-β recommends that ASC pro-inflammatory conditioning strengthens their immunoregulatory properties [54].

Chemokines play important roles in the recruitment of leukocytes leading to various immune responses [118, 119]. The neutrophil, monocyte and eosinophil chemoattractants, at mRNA levels, including chemokine C-X-X motif ligand *(CXCL)-1* and -*6* were increased in ASCs cultured with MLR, whereas, the genes of T- lymphocytes attractants including *CXCL-9, CXCL-10, CXCL-11* were upregulated in ASCs treated with IFN-ɤ, TNF-α, and IL-6 [14]. Individual IFN-ɤ priming enhanced the secretion of chemokines such as monocyte chemotactic protein 1 (MCP-1) and human interferon-inducible protein 10 (IP-10)/ CXCL-10 [61], IL-8 and CCL-5 [83] or CXCL-9, CXCL-10 and CCL-8 [85] by treated ASCs, promoting immune cells recruitment to their close proximity to exert immunomodulatory functions [61, 120]. CM of TNF-α treated hASCs has also been reported to promote monocyte migration in vitro via the enhanced secretion of IL-6, IL-8, CXCL-6, CXCL-2, and MCP-1/CCL-2 (chemokine C–C motif ligand 2) [102]. The synergistic treatment with TNF-α and IL-1β enhanced the expression, by MSCs from different sources including AT, of a number of ECM proteins and chemokines including, among others, CXCL-2, CXCL-6, IL-8, CCL-2 [121].

An additional boosting strategy is to precondition ASCs with TLR agonists. TLRs are members of a large family of receptors (e.g. TLR1-10), among which TLR3 and TLR4 are highly expressed by human MSCs [126]. In the context of treatment of hASCs with lipopolysaccharide (LPS)/TLR-4 agonist, it has been reported that hASCs retained short-term memory when exposed to TNF-α or LPS. Transient treatment with TNF-α or LPS dramatically increased the release of IL-6, IL-8, and MCP-1, and all cytokine levels remained elevated, even after re-plating and culture of hASCs in the absence of stimulating factor. A second round of stimulation induced quick secretion of the cytokines. Importantly, LPS-primed ASCs displayed enhanced therapeutic efficacy in skin flap survival in a diabetic rat model than did unprimed ASCs. Three miRNAs (mir-146a, mir-155, and mir-150) and 5 hydroxymethyl cytosine, epigenetic regulatory molecules, mediated the observed short-term memory of hASCs to LPS or TNF-α [124].

Some studies have suggested that LPS promotes ASCs to acquire a pro-inflammatory phenotype [123, 125], however, the immunomodulatory functions of LPS-ASCs in coculture with activated immune cells in such studies were not tested. Models of acute or chronic inflammation of hASCs were established by treatment of cells with 1 μg/mL LPS for 6 h or 4 weeks, respectively [125]. LPS activated the TLR4/TLR2/NF-κB/ STAT3 signaling pathway to produce the inflammatory cytokines IL-6, IL-1β, and TNF-α via most likely downregulating miR-223. LPS also significantly down-regulated the expression of miR-2909, to upregulate its target, the transcription factor; Kruppel-like factor 4 (KLF4) which in turn significantly, in the presence of activated NF-κB, upregulated the indicated pro-inflammatory cytokines expression. The authors demonstrated that miR-223 and miR-2909 play important roles in the immune-regulatory activity of hASCs, forming a complex regulatory network with pro-inflammatory factors and signaling pathways in ASCs stimulated by LPS [125]. The discrepancy of LPS-ASC immunoregulatory role from immunosuppressive to immunosupportive may be attributed to the differential LPS concentration and treatment duration [131].

Many reports demonstrate that priming of MSCs with the TLR3 agonist, Poly I: C promotes their anti-inflammatory phenotypes and functions [132, 133]. In the context of hASCs and Poly I: C treatment, studies have been conducted and inconsistent effects have been reported [78, 85, 108, 122, 126, 128]. Some authors revealed the expression of several immunosuppressive and inflammatory cytokines by Poly (I:C)-hASCs [126, 128], while, others reported that TLR3 signaling in ASCs did not significantly influence their immunoregulatory phenotype [85, 108, 122]. More studies are thus needed to investigate the immunosuppressive functions of TLR3-primed ASCs in vitro to drive the effective Poly I: C concentration. TLR3 priming of ASCs to promote their Treg-mediated generation as earlier demonstrated with BMSCs [134], can be considered as an interesting research. The synergistic effect of incubation of hASCs with a combination of a pro-inflammatory cytokine and one of the TLR agonists should also be studied.

### Hypoxia Preconditioning

Hypoxia preconditioning generates ASCs with improved therapeutic immunosuppression [68, 71, 101, 130]. One of the suggested mechanisms that mediate the enhanced MSC biology and function in hypoxia is the induced expression of hypoxia inducible factor 1 alpha [100, 135] and the production of various growth factors, such as vascular endothelial growth factor (VEGF), fibroblast growth factor (FGF), platelet-derived growth factor (PDGF), hepatocyte growth factor (HGF), or insulin-like growth factor (IGF) [9]. Additionally, hypoxic ASCs were able to upregulate strongly the expression of the immunomodulatory molecules IDO and PD-L1 upon stimulation with IFN-ɤ and TNF-α [71]. Hypoxia also maintained the chemoattractive properties of ASCs, as evidenced by the enhanced expression of the lymphocytes attractant CXCL-10 [71]. In another report, dual priming of hASCs with IFN-ɤ and hypoxia potentiated their T cell inhibition and the underlying mechanisms were unraveled to be the augmented expression of IDO, PD-L1 and HLA-G via IFN-ɤ and the hypoxia-mediated shift of the hASC metabolism to glycolysis, causing rapid glucose turnover and production of T-cell inhibitory lactate levels [130]. An advanced in vitro culture system for maintained constant O2 levels is crucial to ensure quality-controlled hypoxia-treated ASCs, which would contribute to reproducible results [101].

### Chemical or Pharmacological Preconditioning

Different chemical treatments potentiate the immunosuppressive phenotype and/or action of hASCs on immune cells [136–141]. High concentrations of nanocurcuminoids (12–100 µM) enhanced the expansion and the frequency of CD4Tregsin PBMCs in coculture with hASCs. Additionally, nanocurcuminoids at low doses (below 12 µM) in ASCs/PBMCs cultures were able to decrease the expression of the inflammatory cytokines, IL-17, IFN-ɤ, and IL-6. As well, low doses of nanocurcuminoids augmented the antioxidative capacity of hASCs, as manifested by increased superoxide dismutase activity [136]. Interestingly, priming of ASCs with curcumin before cryopreservation potentiated the viability and functional potency of thawed ASCs [142]. In another report, treatment of hASCs with the active form of vitamin B6, pyridoxal-5'-phosphate (PLP) (at a concentration of 50 ng/ml) enhanced their immunosuppressive effect on CD3^+^ CD8^+^ T lymphocytes via activation of IDO mediated- Trp metabolism and promotion the accumulation of kynurenine (a Trp catabolic metabolite). Specific blocking of TLR4 reduced CD3^+^ CD8^+^ T lymphocytes inhibition by 50 ng/ml PLP-treated hASCs indicating the involvement of the TLR4/NF-κB axis in the PLP-stimulation hASC immunomodulation [137].

Treatment of MSCs, from BM and AT, with specific epigenetic regulatory modulators such as 5-aza-2-deoxycytidine (5-aza-dC) could modulate their immunoregulatory capability via upregulating the mRNA expression of the immunomodulator, HLA-G [138]. Another study reported that preconditioning of hASCs with the iron chelator deferoxamine (DFX), a hypoxia mimetic agent, induced a significant increase in the ASC secretion of anti-inflammatory factors as IL-4 and IL-5 [139]. Importantly, incubation with astragaloside IV, a traditional Chinese medicine at 30 or 60 μg/ml restored significantly the expression of *PD-L1* and *TGF-β* and attenuated the expression of *IFN-ɤ* by hASCs from patients with psoriasis vulgaris [140]. Short-term incubation times to include 24 h [136, 140], 48 h [137, 139] or 72 h [138], have been reported. However, no study addressed the challenge time as a determinant for effect of the applied pharmacological or chemical treatment on ASC immunosuppressive phenotype or action. Recently, long term (14 days) treatment of hASCs with Naltrexone hydrochloride (NTX), an antagonist of mu-, delta-, and kappa-opioid receptors, upregulated the IL-6 secretion by hASCs and promoted the induction of IDO and *PD-L1* in IFN-ɤ treated hASCs. Such effects proportionated directly with the NTX concentration [141]. Treatments of hASCs with cannabinoid compounds have been reported to protect the cells from tunicamycin-induced endoplasmic reticulum stress and inflammation, which resemble those associated with metabolic and inflammatory diseases [143, 144]. However, the stimulating effect of those compounds on hASC immunosuppressive functions in vitro has not been tested. In conclusion, the above-mentioned studies recommend priming of hASCs with chemical or pharmacological molecules could augment their immunosuppression and/or oxidative stress resistance in the settings of inflammatory diseases.

### Genetic Engineering

Direct transduction of immunomodulators and anti-inflammatory factors in ASCs by gene editing may represent a promising approach to enhancing the immunosuppressive functions of ASCs**.** Among the immunoregulatory factors have been delivered in human ASCs are IL-4 [145], fusion proteins comprising the extracellular domain of cytotoxic T-lymphocyte-associated protein 4 and the CH2-CH3 domains of immunoglobulin (CTLA4Ig) [146], glial-derived neurotrophic factor (GDNF) [38], TGF-β1 [147], or HLA-G1 [148]. In vitro cultured GDNF-ASCs induced a shift in macrophage phenotype from the inflammatory (M1) phenotype to the reparative (M2) phenotype [38]. TGF-β1 transduced-ASCs displayed strong IFN-γ-mediated immunosuppressive. ASCs overexpressing TGF-β1 significantly upregulated the expression of IL-10 in CD4^+^ T cells and downregulated the expression of IL-17A, IL-21, and IL-22 [147].

## ASC-related Parameters

### Anatomical AT Harvest Site

For developing effective ASC immunotherapy, it is important to consider the effect of fat depots. Whether derived from visceral (v.) or subcutaneous (sc.) AT, ASCs have differential biological characteristics, metabolic properties, multipotency [149, 150], and response to inflammation [151]. ASCs exhibit depot-specific gene expression profiles [152]. Additionally, within the sc. AT, the harvesting area is a strong determinant of the quality of hASCs, influencing cell viability and yield [153]. Limited studies investigated the immunomodulatory phenotype [154, 155] and/or functions of ASCs [156, 157], derived from different origins under the same experimental setup. Serena et al. reported that CM from healthy sc. ASCs, with normal weight, effectively suppressed activated T cell proliferation, however, that of healthy v.ASC failed to do. CM from sc.ASCs or v.ASCs from patients with T2D and obesity did not attenuate T cell proliferation. Interestingly, v.ASC reflected the inflammatory status associated with the metabolic disturbances (T2D and obesity) than sc.ASCs did, by expressing higher levels of IL-1β, IL-6, MCP-1, TNF-α, inflammasome components and caspase-1 [156]. Further, CM of superficial and deep sc.ASCs promoted equally the polarization of M2 anti-inflammatory macrophages in THP-1 monocytes, possibly via PGE2 and TSG-6 dependent mechanisms [157]. Omentum ASCs have been reported to have a secretome with enhanced anti-inflammatory capacity and higher cytokines levels, except for IL-8, relative to that of sc.ASCs, despite being with lower yield [155]. In another report, a comparative analysis for the expression of immune-related surface and soluble markers by sc.ASCs isolated from two different anatomical locations, (abdomen vs. breast), from different donors, was performed [154]. The results revealed a significant elevation in the expression of the two potent immunosuppressive genes, *IL-10* and *IDO* as well as the expression of the multifaceted immunomodulatory adipokine, visfatin, in breast vs. abdominal ASCs. Such data shed light on the possible therapeutic applications of breast ASCs in inflammatory diseases [154]. Conclusively, the fat depot location, whether subcutaneous or visceral or subcutaneous from different anatomical sources, may impact the immunomodulatory properties of hASCs.

### ASC Differentiation

Some studies addressed the immunogenic and/or immunosuppressive properties of hASC-differentiation derivatives [79, 158–162]. Osteogenically differentiated hASCs and BMSCs retained similarly low expression of HLA-DR and costimulatory molecules (CD40, CD40L, CD80, and CD86) [79]. They did not induce proliferation of HLA mismatched PBMCs in MLR, even after induction of HLA-DR expression via licensing with IFN-ɤ and TNF-α [159]. Addition of biomaterials that stimulate bone tissue formation did not alter MSC immune-related properties [159]. In the context of the chondrogenic lineage, ASC- and BMSC- derived chondrocytes retained hypoimmunogenicity, as manifested by non-induction of PBMC alloproliferative response, even after licensing of MSCs with the inflammatory cytokines IFN-ɤ and TNF-α [160]. Both MSC-derived chondrocytes displayed dose-dependent immunosuppressive functions on T cells and NK cells. Interestingly, ASC-derived chondrocytes, but not BM-derived respective, inhibited strongly allostimulated PBMCs at low cell dosages, suggesting that ASCs would be better than BMSCs for cartilage repair. Noteworthy that the maintained immunoregulatory potential of ASC-derived preosteoblasts [159] or chondrocytes [160] was mediated partly via the expression of HLA-G5 and its level was boosted in immune-active environments. In another study, chondrogenic differentiation or IFN-ɤ treatment potentiated the immunosuppressive effects of hASCs on mitogen-treated PBMC activation and proliferation via secreting expression of higher levels of IL-10 and the surface immunomediator, jagged-2 [161].

Moreover, adipogenic differentiation of hASCs kept their ability to inhibit neutrophil and lymphocyte recruitment to TNF-α-treated endothelial cells in an IL-6/suppressor of cytokine signaling 3 (SOCS3)-dependent manner [162]. On the contrary, adipocytes derived from BMSCs lost their immunoprotective effects on neutrophils, but not lymphocytes. The authors proposed that soluble bioactive molecules, generated by BMSC‐derived adipocytes in coculture with the target immune cells, induce a reduction in TGF-β1 response, modulating IL‐6 signaling, such that it is no longer immunoprotective. Abnormal adipogenesis of MSCs in inflammation thus adversely affect MSC behavior; loss their immunosuppressive effects and contribute to the pathogenic recruitment of leukocytes [162]. It has been reported that adipocytes, like their progenitors, from obese donors promoted the IL-6 mediated-Th17 differentiation in vitro (in coculture with PHA-PBMCs) and in a murine model [163]. Similarly, adipocytes differentiated from sc.ASCs or v.ASCs from T2D patients exhibited elevated IL-1β phenotype as their precursors [156]. Further studies to explore the immunogenicity and immunoregulatory properties of ASC-derived cell products in health and disease may be a crucial step, beside the cell product functionality, to develop an effective applicable ASC derivatives-based therapy.

### ASC Culture Conditions

Diverse culture conditions could impact variably hASC immunomodulation, including among others the serum supplement of the culture medium [17, 53, 164, 165], suspended spheroid vs. mono-adherent layer culture [86, 166], and passage number (early vs. late) [13, 52, 167–170].

#### Serum Supplementation

To date, most studies use fetal bovine serum (FBS)—based culture media to address the immunomodulatory functions of hASCs in vitro. However, safety concerns have been raised regarding FBS addition for the manufacturing of clinical grade MSC products [171], most of them related to immunological risk or possibility of disease transmission due to prions, bacteria, or viruses [172]. Regulatory-complaint xeno-free media such as chemically defined media [173], or media supplemented with human serum (HS) [67], or human platelet lysate (hPL) [173, 174] are promising alternatives to preserve or even enhance the ASC immunomodulatory functions in vitro.

Patrikoski et al. [53] assessed the immunogenic and immunosuppressive properties of hASCs expanded in serum and xenobiotic-free medium (SF/XF) or medium supplemented with FBS or HS. hASCs expanded in any of the three conditions did not lose their hypoimmunogenicity and they were able to suppress the proliferation of PBMCs in two-way MLR. However, the significantly strongest suppression was observed by those expanded in FBS and such effect was attributed to the higher expression of ICAM-1 and IL-6 [53]. In another study and relative to those expanded in FBS, platelet poor Plasma (PPP)—cultured ASCs exhibited compromised potential to generate Tregs and that was correlated with limited PPP-ASC potential to produce soluble immunomodulatory factors [165]. In contrast, PPP supplementation promoted the expression of vascular cell adhesion molecule-1 (VCAM-1)/CD106 and ICAM-1 on ASC surface hinting possibly toward maintained direct immunosuppressive mechanisms. These data confirm the strong effect of culture media composition on ASC immunomodulatory behavior as well as serving as an alert regarding the complexity of replacing FBS in MSC culture [53, 165].

Other authors reported enhanced or at least maintained hASC immunosuppressive functions in the absence of FBS [12, 67, 173, 175]. Effective inhibition by hASCs on CD4 T cell proliferation, activation, and functions in a 5% HS-supplemented culture medium, have been recently reported [67]. As other alternatives, ASCs isolated from human infrapatellar fat pad (hIFP-ASCs) were processed in the presence of hPL, chemically reinforced medium (Ch-R), or FBS [173]. hIFP-ASCs cultured in the regulatory-compliant conditions (hPL or Ch-R) displayed enhanced anti-inflammatory surface and paracrine phenotype which was intensified by priming of hIFP-ASCs with IFN-ɤ and TNF-α. In the two indicated regulatory-compliant conditions, hIFP-ASCs upregulated CD10/Neprilysin expression which degraded substance P in vitro and in vivo, relieving experimental synovitis [173]. Interestingly, hASCs expanded in clinical grade hPL were more potent in inhibiting T-cell growth, than BM counterparts via a superior IFN-ɤ-mediated IDO activity [175]. Additionally, resting and inflammatory primed ASCs-PL expressed higher transcriptional levels of TSG-6 [12], which is a suppressor to neutrophil recruitment in acute inflammation [176]. In another report, fabricated hASC sheets in the presence of human PL exhibited enhanced deposition of ECM and inhibition of stimulated macrophages migration [177]. hPL- ASCs are thus suggested as interesting anti-inflammatory cell therapy for further preclinical and clinical evaluations.

#### Three-Dimensional ASC Cultures

The traditionally cultured MSCs in a two-dimensional (2D) adherent monolayer exhibit limited physiological relevance and altered genetic and epigenetic signatures [178]. Thus, cultivation of MSCs in three-dimensional (3D) systems, such as low attachment surfaces, hydrogels, or scaffolds, to resemble the in vivo spatial organization with increased cell–cell and cell–matrix interactions, have gained attention [171, 179]. The methods to generate 3D MSC spheroids, including ASC ones, and the limitation and challenges of different MSC spheroid generation platforms have been recently reviewed [171, 180].Enhanced therapeutic and anti-inflammatory mechanisms have been reported in different preclinical models treated with human ASCs cultured by a 3D strategy [86, 100, 178, 181–190].

##### Scaffold-free 3D ASC Spheroids

Limited studies addressed the immunomodulatory phenotype and/or functions of scaffold free- 3D ASC spheroids in vitro [59, 86, 166]. hASC aggregates, obtained by the hanging drop technique, exhibited a stronger ability than 2D counterparts, to modulate macrophage polarization from M1 (pro-inflammatory) to M2 (anti-inflammatory) phenotype in vitro, presumably via elevated PGE2 production [86]. Recently, ASC spheroids significantly abrogated the proliferation and IFN-ɤ secretion and promoted the production of the anti-inflammatory cytokines IL-9 in coculture with stimulated γδ CD3^+^ T lymphocytes [59]. γδ CD3^+^ T lymphocytes represent a bridge b/w innate and adaptive T cell responses and produce high levels of IL-17 in obesity contributing to AT inflammation and insulin resistance [77]. The detected anti-inflammatory effects were greater than those observed by adherent ASCs due to higher levels of the immunomodulators IL-5, IL-10, IL-4 and IL-13 in the ASC spheroid secretome [59]. In another report, comparative gene expression analysis in 2D vs. 3D (in ultra-low attachment surfaces) ASC cultures revealed superior expression, of genes involved in stemness (*SOX2, POU5F1,* and *NANOG*), anti-aging (*SIRT1*), and anti-inflammation (*TGF-β1*), however, lower expression of those involved in oxidative stress (*ALDH3*), in 3D ASC spheres [166].

##### Scaffold-based 3D ASC Spheroids

Some authors addressed the immunomodulatory properties of hASCs encapsulated in hydrogels [185, 188, 191]. hASCs encapsulated in injectable alginate hydrogel modified with arginine-glycine-aspartate- motifs did not induce DC maturation and they exhibited potentiated inhibitory lymphocyte proliferation. Importantly, hASCs in alginate responded like hASC monolayer to IFN-ɤ licensing [185]. Such results indicate that the combination of ASCs and alginate is non-immunogenic, however, immunosuppressive. That might be attributed to the elevated secretion of the chemokine IL-8 by 3D ASCs in inflammation [185]. As well, incorporating the hASCs spheroids in alginate microbeads in thermosensitive hydrogels [188] enhanced significantly the expression of immunosuppressive cytokines such as IL-10 and/or TGF-β1 for controlling inflammation in wound healing. As well, hASC-spheroids in non-crosslink hyaluronic acid gel (4%) displayed a promoted expression of some angiogenesis growth factors, pluripotency markers, the anti-inflammatory factors (*IL1RN, IL11,* etc.), in addition to compared to the adherent ASC cultures [191]. Such studies recommend that aggregation of ASCs in hydrogels stimulates paracrine signaling and the level of anti-inflammatory factors [19], and potentiates the cell functionality [192]. The positive effects can be amplified by incorporating of the ASC spheroids in tunable immunotolerant polymeric hydrogels to tailor the target therapeutic effects [193]. Interestingly, sustained MSC licensing could be achieved by the chemical modification of the hydrogel capsule to present an inflammatory cytokine [194] or by incorporating the cytokine in the hydrogel matrix [193]. Such strategies might ensure enhanced MSC persistence and immunomodulation in vivo.

Anoikis is the apoptosis of adherent cells due to the lack of a scaffold, so the cultivation of MSCs on a fabricated scaffold can prevent it [195]. However, matrix stiffness (mechanical properties), construct dimensions, fiber alignments, and/or fluid forces may affect ASC characteristics [196, 197] including immunomodulatory ones [198, 199]. Wan et al. studied the effect of fiber orientation (random or aligned), as one of the physical features of the scaffold, on the ASC immunomodulatory paracrine mechanisms. hASCs seeded on aligned fibers secreted significantly higher levels of immunomodulators, including among others, COX-2 and TSG-6, than those cultured on random fibers and that was correlated with a superior promotion of M2 macrophages. Aligned fibers stimulated ASC immunomodulatory function by activating mechanotransduction pathways; focal adhesion kinase (FAK)-extracellular regulated kinase 1/2 (ERK1/2) and YAP/TAZ [198]. FAK has been reported to mediate the cellular responses to the biomaterial physical cues [200, 201], and has been directly linked to transcriptional regulation of COX-2 [202]. The inhibition of YAP/TAZ nuclear translocation reduced the gene expression of crucial immunomediators including *COX-2, TSG-6, IL-1RA, and MCP-1* in hASCs cultured on aligned fibers [198]. Signaling mechanisms regulating the MSC response, specifically those derived from bone marrow, to the physical cues of scaffolds are recently reviewed [199]. In the context of hASCs, further transcriptomic, proteomic and functional studies to address the immunomodulatory capacity of scaffold-free- or based- hASC spheroids are demanded.

### ASC Passaging and Cryopreservation Effects

Expansion of hASCs till passage 6 (P6) P6 did not reduce the immunomodulatory properties of hASCs, whereas, cryopreservation significantly did [52]. Analysis of hASC immunophenotype including the immuneregulatorymarkers;CD200, CD274, CD271, CD73, and CD29 [203, 204] over 8 passages revealed that hASCs maintained the expression of these markers at variable levels over the whole culture period without significant differences except for CD271 which decreased by culture [170]. On the other hand, a larger literature cohort recommends that only ASCs of low passage rounds would be ideal for immunomodulatory therapeutic purposes [13, 167–169, 205]. ASCs at late passages failed to inhibit IFN-ɤ production by PBMCs and to abrogate neutrophil activity and the levels of pro-inflammatory markers TNF-α and IFN-ɤ in a model of peritonitis [168]. In another report, ASCs at P3 downregulated the proportion of Th17 cells, in patients with active systemic lupus erythematous, and their abilities to produce IL-17, whereas ASCs at P8 had a contrasting effect [205]. hASCs at late passages had reduced levels of secreted IL-10 and HGF [168], in addition to the surface (CD200 and CD274), or intracellular (heme oxygenase 1 (HO-1)), proteins [13], and all these factors contribute to effective ASC immunomodulation[19]. Decreased gene expression of the anti-inflammatory factors (TSG6 and HLA-G) was also detected in ASCs and BMSCs over repeated passages up to P10 [169]. Regarding the expression of MHC proteins in repeatedly passaged ASCs, HLA-ABC (MHC-1) level was not affected by expansion. However, levels of HLA-DR and the surface and the intracellular HLA-G in ASCs decreased by expansion [13].

Overall, in vitro-aged hMSCs, as a result of extensive culture expansion, have been reported to show senescence signatures, diminished immunosuppressive capacity, and weakened regenerative potential as well as pro-inflammatory features [206]. Impaired autophagy and altered epigenetics molecular mechanisms involved in MSC aging in vitro and in vivo have been reviewed [207].

In clinics, modest immunomodulatory activity of freeze-thawed, relative to fresh, MSC products has been reported [208]. Cryopreserved MSCs exhibited attenuated immunosuppressive properties in vitro as a result of heat-shock response, impaired IFN-ɤ treatment response [209], and T-cell mediated cytolysis [210]. In the context of hASCs, different cryoprotectants have been tested to include dimethyl sulfoxide (DMSO) alone or in combination with pentaisomaltose [211], intracellular delivered trehalose [212]. For clinical grade cryopreservation, xeno and/or DMSO free-cryopreservation solutions containing trehalose, dextran 40, propylene glycol, glycerol, polyvinylpyrrolidone, hydroxyethyl starch, and/or methylcellulose have been developed [213–219]. Despite the diverse protocols [214, 220], up to our knowledge, the impact of cryopreservation and freeze-thawing on the immunomodulatory properties of hASCs is still not explored. Thus, future studies, aim to investigate the inhibitory effects of long-term cryopreserved hASCs, using different cryoprotectants, on T lymphocytes and their IFN-ɤ licensing response, are recommended.

## Donor-related Parameters

### Age, BMI, and Sex

Increasing donor age negatively impacts the biological features of hASCs, including decreased expansion kinetics, differentiation potential [221, 222], and/or regenerative capacity [223]. Regarding the immunomodulatory properties, young rat ASCs [224] and canine ASCs [225] exhibited lower antiproliferative effects on activated T lymphocytes than old respective. Recently, an inflammatory state, characterized by elevated expression of *IL-6, IL-1β, TNF-α*, and *MCP-1*, has been detected in hASCs derived from elderly subjects (≥ 65 years) and that was associated with enhanced glycogen storage and decreased expression of sirtuin 1 and 6 [226]. Sirtuins are key metabolic sensors that links inflammation and metabolism [227]. Obesity exacerbated the inflammatory phenotype of elderly hASCs [226].

In the context BMI, numerous investigators reported the adverse effects of obesity [BMI ≥ 30] on the immunomodulatory phenotype and functions of hASCs [39, 77, 156, 226, 228–238]. ASCs isolated from patients with obesity exhibited altered glucose metabolism [226, 239, 240], reduced sirtuins expression [226, 241], pro-inflammatory phenotype [20, 156, 228, 229]. The pro-inflammatory state of hASCs from obese patients characterized by the elevated secretion of inflammatory cytokines and chemokines including IL-6, IL-1β, TNF-α, IL-17A, IL-8 and /or MCP-1 [39, 156, 228, 229, 242]. In addition to the activation of inflammasome components, NF-Kβ [156], and mitogen-activated protein kinase (MAPK) signaling that are linked to inhibiting insulin signaling [77].

In vitro, hASCs from patients with obesity failed to inhibit T cell proliferation [156] and promoted Th17 differentiation via IL-1β [242] and/or PD-L1 [234]-mediated manner. As well obese ASCs potentiated monocyte polarization toward the inflammatory macrophages (M1) [39]. Noteworthy, obesity deteriorates the response of hASCs to IFN-ɤ -induced expression of IDO and that might explain the dysfunctional antiproliferative effect of obese ASCs on T cells [232]. A new mechanism has been recently introduced to explain the reduced therapeutic efficacy of ASCs derived from obese subjects [243]. The decrease in mitochondrial cardiolipin content in obese ASCs resulted in compromised cell potential to sequester their damaged mitochondria into LC3-dependent autophagosomes. That led to mitochondrial dysfunction, impaired intercellular mitochondrial transport, and finally attenuated therapeutic efficacy of obese ASCs. Importantly, pharmacological treatment using a compound that modulate mitophagy and/or autophagy such as pyrroloquinoline quinone rescued the mitochondria health in obese ASCs [243]. Interestingly, the addition of n-3 polyunsaturated fatty acid precursor, alpha-linolenic acid (ALA), or its derivatives, eicosapentaenoic, or docosahexaenoic acid, to co-cultures of human obese ASCs and PBMCs abrogated the immunostimulatory impact of obese ASCs. ALA inhibited obese ASC-mediated activation of Th17 cells, IL-17A secretion, Cox-2 and STAT-3 expression [244]. Another approach to rescue obese ASC biology, cytosol transfer from control ASCs derived from patients with normal weight restored the glucose metabolism in ASCs from patients with obesity via Lin28-mediated repression of let7 pathway [239].

On the other hand, limited studies illustrated maintained or slightly affected immunosuppressive potentials of ASCs from obese subjects [67, 231, 235]. The discrepancies b/w studies on the impact of obesity-associated inflammation and metabolic abnormalities on the immunosuppressive potential of hASCs could be due to differences in fat depots locations, the methods used to evaluate immunosuppressive functions in vitro, or to the use of donors with different adiposity grades, or at different stages of obesity development. Studies that investigated the effect of obesity on hASC immunomodulation are presented in Table 2.

**Table 2:** Studies investigating the immunomodulatory properties of hASCs isolated from donors with obesity

Ref	AT site location	Age (years) (range or mean ± SEM)	Metabolic Indices (Values in range or mean ± SEM)	BMI( kg/m2) (Range or mean ± SEM)	No. of Obese Subjects	The studied immune-related properties and/or functions	The key immunomodulatory properties- relevant findings
[228]	ND	41.56 ± 3.07	Elevated glucose and total cholesterol/high-density lipoprotein (HDL) ratio as compared to non-obese individual (142.13 ± 11.95 mg/dl vs 61.54 ± 7.35 mg/dl) and (5.92 ± 0.48 vs 3.98 ± 0.38), respectively	44.44 ± 1.29	ND	Transcriptome analysis (including inflammatory genes)	From a transcriptome perspective, obese ASCs exhibited reduced stemness, adipogenic commitment, and enhanced inflammatory state (↑*IL-1β, IL-8,* and *MCP-1/CCL-2*)
[242]	v. AT and sc.AT	ND	ND	ND	ND	T cells differentiation	Obese not lean ASCs promoted Th17 polarization in IL-1β, not IL-6,—dependent manner, however, they reduced Th1 cytokines. Increased IL-17A production inhibited adipogenesis and promoted insulin resistance in adipocytes
[229]	sc.AT	37.3 ± 13.4	ND	46.2 ± 5.1	*n* = 12	Cytokines released in the secretome of unprimed and LPS (0.5µg/ml) -stimulated ASCs	Enhanced inflammatory state of obese ASCs (↑IL-6 and IL-8 in unprimed and primed obese ASCs and MCP-1 in primed obese ASCs)
[156]	v. AT and sc.AT (abdomen)	39 ± 8.9	-HOMA-IR 2.84 ± 0.2	33.1 ± 2.1	*n* = 4	-Basal expression of pro-inflammatory cytokines as IL-1β, TNF-ɑ, MCP-1 and critical inflammasome components and anti-inflammatory cytokines as TGF-β1 and IL-10 by obese vs lean ASCs -The potential of CM of obese vs lean ASCs to suppress the proliferation of PBMCs in MLR	- Enhanced inflammatory state of obese ASCs (↑*IL-1β, IL-8, TNF-α,* and *CCL-2*) -As well, obese ASCs exhibited impaired antiproliferative effect due to inflammasome activation-mediated IL-1β elevation, however, reduced expression of TGF-β1
[230]	sc.AT (abdomen)	42.5 ± 8.9	ND	32.7 ± 3.7	*n* = 6	-Potential to suppress murine T cell proliferation The therapeutic efficacy in EAE	-Obese ASCs failed to halt the disease progression in CNS of EAE -Obese ASCs enhanced proliferation and differentiation of CD4 and CD8 T cells
[231]	sc.AT (abdomen)	18–62	ND	Morbidly obese, however, exact BMI is ND	*n* = 10	- Potential to suppress CD3 T cells proliferation Isolated from RA patient -induction of Tregs -Modulation of cytokines production	Morbidly obese ASCs were able to inhibit proliferation of CD3 T cells, derived from RA patients, in a dose-dependent fashion, however, they promoted that of CD4^+^ FOXP3^+^ T cells**.** They also downregulated the production of IFN-ɤ and TNF-ɑ, however, upregulated IL-10 by RA-CD3 T cells
[241]	v. AT and sc.AT	40.40 ± 1.14	HbA1c (%): 5.46 ± 0.28 vs 4.70 ± 0.43 CRP: 1.23 ± 0.19 vs 0.37 ± 0.11 in obese vs non-obese individual	44.66 ± 4.64	*n* = 8	-Gene expression of Sirtuins 1–7 in naïve and hypoxia primed sc.ASCs ss v.ASCs	Sirtuin1–6 mRNA levels were markedly reduced in v.ASCs of obese patients. Sirtuins’ expression in v.ASCs correlated negatively with BMI and CRP. Hypoxia-induced mRNA expression of all of the sirtuins only in obese v.ASCs
[20]	Sc.AT	35–45	ND	≥ 30	*n* = 5	-Assessment of intracellular vitamin D levels - Gene expression of pro- and anti-inflammatory mediators	Expression of *IL-6, IL-8, IL-10* and *MCP-1* was higher in obese ASC than in control ASCs and that was associated with reduced vitamin D levels
[233]	sc.AT (abdomen)	40.50 ± 7.46	ND	33.97 ± 3.11	*n* = 6 (pooled)	-The potential to modulate macrophage and microglia polarization toward anti-inflammatory phenotype -Modulation of gene expression of pro- and anti-inflammatory markers by macrophages -Modulation of NO activity and phagocytic activity of macrophages	Obese human ASCs induced polarization of murine microphages and microglia toward a pro-inflammatory phenotype
[234]	v. AT or sc.AT	ND	ND	> 30	ND	The contribution of PD-L1 to the obese ASC-mediated induction of Th17	Inflammation, mediated by ASCs from obese individuals, contributed to PD-L1 upregulation in cocultures with PBMCs (non-activated or PHA-activated) Blocking PD-L1 expression in cocultured cells (ASCs and PBMCs) restored TNF-α and IL-2 expression by Th1 cells and improved obese ASC-induced Th17 cell activation, without affecting pro-Inflammatory cytokine secretion by accessory cells
[235]	sc.AT	33.5–34.1	-HOMA-IR range 0.6–2.7 -Plasma adiponectin: 1310–4930	25.2–44.2	*n* = 5	-Immunogenic effect -Suppression of PBMC proliferation in MLR	Leaner and heavier WD donors were hypoimmunogenic, however, heavier WD donors showed superior immunosuppressive capacity
[39]	ND	57.0 ± 2.6	50% of obese subjects had hypertension, 66% had T2D, and 66% suffered from hyperlipidemia	42.9 ± 1.1	*n* = 6	Potential to modulate the polarization of macrophage in inflammation toward anti-iinflammatory phenotype (M2) at the expense of inflammatory phenotype M1	-ASCs isolated from obese donors had blunted immunomodulatory potential on activated macrophages - TNF-α levels were four-fold higher in CM collected from obese than from lean ASCs
[236]	v.AT	45.7 ± 6.7	ND	31.4 ± 1.8	*n* = 11	- Potential of the secretome of obese or lean v.ASCs to modulate macrophages in the tumor microenvironment	Only obese v.ASCs could modulate macrophage to acquire pro-tumoural phenotype, characterized by the expression of pro- and anti-inflammatory phenotype. That was mediated via internalizing the obese ASC adipokine, survivin by the macrophages, and inducing survivin expression by the tumor-associated macrophages
[226]	sc.AT	Adult group (48.7 ± 8.5) Elderly group 72 ± 4.7	Obese and lean groups exhibited comparable glucose levels and lipid profile	Adult group 34.3 ± 4.5 Elderly group 33.3 ± 3.5	Adult group *n* = 30 Elderly group *n* = 8	- Multiplexed cytokine array (62 cytokines) - Gene expression analysis of inflammatory markers and genes involved in glucose metabolism - Lactate secretion and glycogen content analysis - SIRT1 and SIRT6 protein expression	-Aging significantly promoted the inflammatory state of sc.ASCs as characterized by elevated expression of IL-6, IL-1β, TNF-α, and MCP-1 -Aging significantly enhanced glucose turnover, lactate secretion, and glycogen storage -Obesity exacerbated the inflammatory state of ASCs -Reduced expression of SIRT1 and SIRT6 mediated the adverse effects of aging and obesity on the metabolism and the phenotype of sc.ASCs
[237]	ND	39.43 ± 6.97	All the tested biochemical parameters were comparable between the obese and lean subjects, except for a higher cholesterol/HDL ratio in the patients with obesity	44.14 ± 5.46	*n* = 7	- Secreted cytokines profiling	Significant low expression of pro-inflammatory (MIP3a, IL-8, and TNFα) and regulatory (GM-CSF, Fractalkine, IL-6, IL-7, and IL-21) cytokines and chemokines in ASCs derived from obese patients, as compared to those of ASCs derived from healthy donors with normal weight

*AT* adipose tissue, *BMI* body mass index, *CCL-2* C–C motif chemokine ligand 2, *CNS* central nervous system, *CRP* C—reactive protein, *CM* conditioned medium, *EAE* experimental autoimmune encephalomyelitis, *FOXP3* forkhead box P3, *GM-CSF* granulocyte–macrophage colony-stimulating factor, *HbA1c* glycated hemoglobin, *HDL* high-density lipoprotein cholesterol, *HOMA-IR* homeostatic model assessment for insulin resistance, *IL-6* interleukin 6, *IL-1β* interleukin 1 beta, *IL-17A* interleukin 17A, *IL-10* interleukin 10, *IL-8* interleukin 8, *IL-7* interleukin 7, *IL-21* interleukin 21, *MCP-1* monocyte chemotactic protein 1, *MLR* mixed lymphocyte reaction, *mRNA* messenger ribonucleic acid, *MIP3a* macrophage inflammatory protein 3 alpha, *ND* not defined, *NO* Nitric oxide, *PD-L1* programmed death ligand 1, *PBMCs* peripheral blood mononuclear cells, *RA* rheumatoid arthritis, *SEM* standard error means, *s.AT* subcutaneous adipose tissue, *SIRT1* sirtuin 1, *SIRT6* sirtuin 6, *Tregs* T cell with immune regulatory functions, *TNF-ɑ* tumor necrosis factor-alpha, *TGF-β1* transforming growth factor beta 1, *Th17* CD4 T helper 17 cells, *Th1* CD4 T helper 1 cells, *T2D* type 2 diabetes mellitus, *v.AT* visceral adipose tissue, *WD* weight- discordant

Sex impact on the immunomodulatory properties of hASCs has been recently studied [110, 127]. Mckinnirey et al. examined the potency and functionality of both female and male ASCs in order to gain further insights into donor selection. Female ASCs, significantly suppressed activated PBMC proliferation more than male ASCs did, due to the production of higher concentrations of the anti-inflammatory factors; IDO1, IL-1RA, and PGE-2 and the prolonged expression of VCAM-1 post-activation [110]. In another report, inter-individual variability and/or possible sex differences exist in ASCs’ response to LPS treatment and the potential of LPS-ASC CM to regulate CD14 expression in THP-1 human monocytes have been reported [127]. Variables, including age, sex, and biological sources of MSC, that can guide the important choice of “universal” or “personalized” MSC therapy for autoimmune diseases have been recently reviewed [245, 246].

### Presence of Inflammatory Disease

The impact of inflammatory diseases on the immunosuppressive properties of hASCs has been extensively investigated to include RD [46, 47, 96, 112, 231, 247–257], T2D [67, 109, 156, 258], sepsis [259], Crohn’s disease [260–263], ulcerative colitis [264], breast cancer [265–269], osteoporosis [270], psoriasis vulgaris [140], Parkinson's disease [271], and atherosclerosis [272]. The results are contradictory from diseased ASCs having intact immunomodulatory functions to impaired immunosuppressive effects.

#### Effect of RD

The RD include a wide range of auto-immune and inflammatory disorders that affect bone, tendon, ligaments and/or muscles. Examples of RD forms are rheumatoid arthritis (RA), OA, systemic lupus erythematosus (SLE), systemic sclerosis (SSc), and ankylosing spondylitis (AS) [46, 47, 96, 253, 254]. hASCs inhibited the proliferation and the production of inflammatory cytokines (IFN-ɤ, TNF-α, and IL-17) by collagen II -activated CD4^+^ and CD8^+^ T cells from patients with RA. hASCs also stimulated the generation of CD4^+^CD25^+^FOXP3^+^ Tregs, with capacity to suppress collagen-specific T cell responses. Finally, hASCs downregulated the inflammatory response of synovial cells isolated from patients with RA, by downmodulating the production of matrix-degrading enzymes [247]. hASCs were able to modulate Th17 responses by inhibiting the gene expression and/or secretion of IL-17, IL-21, and/or IL-6 by PHA-activated PBMCs isolated from RA vs. healthy donors, with more intense suppressive effects in the RA group due to their priming by the patients’ inflammation. Noteworthy, hASCs remarkably induced TGF-β1 expression in healthy PBMCs [249]. Not only the cells are effective, but also the secretome of healthy hASCs were able to downmodulate Th17 cells and significantly increase Tregs in coculture with PBMCs from RA patients [257].

Regarding the immunosuppressive potential of ASCs isolated from patients with RD, controversial data have been reported from mostly intact [47, 112, 254, 273], to affected [250], ASC immunosuppressive functions. RD/ASCs (from patients with SLE, SSc, or AS) were characterized by low basal levels of CD90 and ICAM-1 expression, upregulated secretion of IL-1Ra, TSG-6 and sHLA-G, but impaired release of kynurenines and galectin-3 [252].

Despite the altered immunomodulatory phenotype, intact immunosuppressive effects of hASCs derived from patients with SLE, SSc, or AS have been reported by the same research group [47]. Comparable to healthy ASCs, RD/ASCs were able to modulate the activation of allogeneic CD4^+^ and CD8^+^ T lymphocytes in direct and transwell coculture settings. RD/ and healthy ASCs attenuated the expression of CD25 and HLA-DR on T lymphocytes, however, upregulated the CD69 level [47].. Recently, ASCs from patients with RA or OA exhibited intact lymphocytes antiproliferative potential via mostly the induced release of IL-10 and PGE2 and the enhanced activity of IDO [254]. In another report, hASCs derived from healthy donors and SSc patients with extracutaneous manifestations presented the comparable potential to inhibit the PHA-activated PBMCs proliferation in direct contact settings [273].

On the contrary, Skalska and Kontny revealed that the immunosuppressive and anti-inflammatory functions of ASCs derived from inflammatory rheumatoid joints of patients with RA or OA are impaired [250]. ILl-17A is one of the crucial mediators in the development of RA [274]. The enhanced release of this cytokine by activated PBMCs after contact with rheumatoid ASCs may recommend their involvement in disease progression by promoting pro-inflammatory activity [250, 251]. Treatment of RA-ASCs with high/moderate molecular weight adiponectin only was found to considerably upregulate the secretion of the soluble factors IL-1RA, PGE2, TGF-β, IL-6, IL-8, and VEGF, however, it did not greatly impact the weak immunosuppressive effects of RA-ASCs on PHA-activated PBMCs [250].

#### Effect of Inflammatory Bowel Diseases

Inflammatory bowel disease (IBD) primarily comprises Crohn’s disease (CD) and ulcerative colitis (UC) [264]. The available studies that addressed the immunomodulatory properties of ASCs from patients with active IBD illustrate that such cells have blunted immunosuppressive functions and so they are not suitable for autologous therapy [261–264]. Serena et al. investigated the altered immune profile of hASCs derived from mesenteric or subcutaneous fat depots from CD patients. Mesenteric creeping fat hASCs, of CD patients, exhibited exacerbated migration capacity and elevated *IL-1β* expression. Also, CM from active and inactive CD subcutaneous ASCs failed to inhibit the proliferation of stimulated T and B cells and to promote the M2 polarization. That was attributed to inflammasome activation and inflammatory markers elevation, as represented by upregulated gene levels of *IL1β, IL6, TNF-*α*A,* and *CCL2*, however, reduced expression and production of the ASC immunomodulator; TGF-β [261]. In a more recent report, the authors demonstrated that sc. ASCs from active CD patients exhibited distinct epigenetic DNA methylation patterns associated with differential expression of immune system- related genes [262]. With except of *TNF-α*, in hASCs isolated from patients with inactive disease, almost the expression levels of all those genes were comparable to the control level, indicating that immune system genes affected by methylation marks were partially restored in patients during episodes of remission [262]. Importantly, cigarette smoking favored the pro-inflammatory epigenetic changes and the blunted immunosuppressive functions of hASCs from patients with active CD [263].

The first report to investigate the immunosuppressive potential of hASCs from UC patients, with different disease degrees has been recently published [264]. UC-ASCs exhibited a diminished ability to inhibit stimulated PBMC proliferation, suppress CD25 and CD69 activation marker expression, decrease the production of IFN-ɤ and TNF-α, and reduce their cytotoxic effect on A549 cells. On inflammatory priming with a mix of IFN-ɤ and TNF-α, UC-ASCs secreted lower levels of PGE2, IDO, and TSG-6, which mediated their blunted immunopotency. Moreover, UC-ASCs induced weaker therapeutic effects than healthy ASCs, in experimental UC. These findings indicate that the immunosuppressive properties of ASCs from patients with UC (mild, moderate or severe) are affected [264].

#### Effect of Breast Cancer

Numerous studies demonstrate that resident ASCs in breast cancer(BC) tissue are greatly affected by the tumor microenvironment [265–269]. In one study, IL-10 and TGF-β1 mRNAs were significantly higher in ASCs isolated from patients with BC (pathological stage II/III) than those from normal individuals. Moreover, the CM of ASCs isolated from patients with BC (stage III) upregulated the expression levels of the regulatory molecules genes; *IL-4, TGF-b1, IL-10, CCR4 and CD25* and increased the frequency of CD4^+^ CD25^+^ FOXP3^+^ Tregs in peripheral blood lymphocytes (PBLs) [265]. In a more recent report, ASCs from BC patients significantly directed naïve CD4 T lymphocytes toward Tregs with different phenotypes. They significantly induced the expansion of the CD4^+^CD25^+^Foxp3^+^CD45RA^+^, CD4^+^CD25^+^ FOXP3^+^Helios^+^, CD4^+^CD25^−^ FOXP3^+^Helios^+^, and CD25^+^ FOXP3^+^CD73^+^CD39^+^ Tregs and that was associated with enhanced production of IL-10 and TGF-β1 by the generated T regs [269].

Another immunomodulatory role seems to be exerted by hASCs in the tumor microenvironment is IFN-ɤ mediated- elevated expression of major histocompatibility complex class I polypeptide-related sequence B (MIC B). MIC B is a ligand of Natural-killer Group 2, member D (NKG2D) receptor to protect the cancer cells from NK cells. Frequent stimulation of NKG2D receptor by MIC B can result in downmodulation of this receptor and the impairment of NK cells activation in invasive ductal breast carcinoma [268]. Another report demonstrated a significant decrease in the percentage of CD3^−^ CD16^+^ CD56^+bright^ and CD3^−^ CD16^+^ CD56^+dim^ NK cell subsets after exposure of PBLs to ASCs either from normal donors or patients with BC (pathological stage II/III). A considerable reduction in NK cell activating receptors as NKG2D and the CD69 among the cocultured PBLs was also observed. However, no significant difference was observed b/w cancerous vs. normal breast ASCs in the NK cell suppressive effects. However, cancerous ASCs had significantly higher *IDO1, IDO2,* and *HLA-G5* mRNAs [266].

In the context of B cells, ASCs from normal donors and patients with invasive ductal BC (stage II/III) were cocultured with B cells derived from breast tumor draining lymph nodes in direct and transwell systems [267]. ASCs from normal donors, not from patients with BC, were able to inhibit proliferation of in direct contact only. However, cancer ASCs induced higher frequency of IL-10 regulatory B cells than normal ASCs did [267]. All available studies recommend that ASCs may have crucial roles in breast tumor growth and progression by inducing regulatory molecules and promoting anti-inflammatory reactions within the tumor microenvironment.

#### Effect of Miscellaneous Inflammatory Diseases

Other different inflammatory or autoimmune diseases may affect the immunosuppressive phenotype and/or functions of ASCs. Under Th17 polarization conditions, hASCs from healthy donors inhibited the differentiation of CD4^+^ T cells, from patients with Parkinson's disease, into Th17 cells, however, they induced functional Tregs producing IL-10Such ASC immunosuppressive findings in Parkinson's disease was attributed to the release of LIF by hASCs [271]. In the context of atherosclerosis (ATH) alone or with T2D, ASCs from patients had compromised ability to suppress the proliferation of activated allogeneic CD4 + T and the effect was more profound in the presence of T2D [272].

In the context of sepsis, three monocyte subsets (CD14^++^CD16^+^, CD14^+^CD16^++^, and CD14^++^CD16^–^) were isolated from patients in the early phase of severe sepsis or septic shock [259].The levels of CD14^++^CD16^+^ monocytes (pro-inflammatory phenotype) were positively correlated with the disease severity scores. hASCs were able in coculture to switch monocytes from CD14^++^CD16^+^ to CD14^++^CD16^–^and modulate the production of inflammatory cytokines toward anti-inflammatory phenotype (increased IL-10 secretion) via PGE2/EP4-dependent mechanism. Additionally, ASCs modified frequenciesof monocyte phenotypes in experimentalsepsis [259]. A research indicates that ASCs, from osteoporotic donors, exhibit superior anti-inflammatory effects, over osteoporotic BMSCs in vitro and in a model of osteoporosis due to their potential to maintain stemness, energy metabolism and anti-oxidative capacity [270].

## Conclusion & Perspectives

In the inflammatory microenvironment, the immunoregulatory potential of MSCs is susceptible to various factors, which leads them either to promote or reduce inflammation. From clinical perspectives, numerous recent review articles presented the effective potential of hASCs in treating of autoimmune/inflammatory or degenerative disorders [1, 4, 19, 275, 276]. Due to their great contribution to hASC therapeutic effectiveness, we discussed the experimental and the donor-related parameters that may affect hASC immunomodulatory functions in vitro. However, contradictory results about the adjustments of most of those determinants have been reported.

It is commonly thought that the younger the donor is, the less the passage of MSCs, the higher their stemness. From multiomics perspective, it has been found that even in ASCs from an elderly subject (≥ 60 years), when the round of cell passages is early, the stemness is high, indicating that ASC passage has a greater impact on the stemness and characteristics of hASCs than donor age [222]. Regarding the experimental setting, direct contact with properly activated immune cells at high cell ratio may ensure effective immunosuppressive potential of hASCs. Regulatory-complaint xeno-free media are promising alternatives to preserve or even enhance the ASC immunomodulatory functions in vitro [67, 173]. However, comparative standardized research focus on profiling the changes in ASC immunomodulation in association with different culture medium compositions, is still needed for efficient FBS alternatives development and safe clinical translation.

ASC priming strategies to enhance their immunosuppressive efficacy are diverse and IFN-ɤ treatment of hASC spheroids seems to be a promising approach. Noteworthy, IFN-ɤ stimulation increased IDO expression noticeably in ASCs over BMSCs and umbilical cord blood-MSCs, however, it did not increase ASC immunosuppressive functions [22]. The latter findings might recommend assessing the IDO activity instead of expression to predict the ASC line immunosuppressive functions. Other licensing conditions can also be applied such as hypoxia, TLR stimulation and pharmacological manipulation. More attention needs to be paid to the affected molecular and signaling mechanisms, in primed ASCs, to personalize the therapeutic outcome and improve the ASC dysfunctionality in disease. Better knowledge of the differences b/w the immunomodulatory potentials of ASCs derived from various fat depots would be of great interest for a better source selection for ASC immunotherapeutic targets. As well, the effect of physical cues such as substrate stiffness and fluid forces in 3D platform on the immunomodulatory capacity of ASCs requires further research in the near future [200].

In the context of the impact of pathological conditions on the immunomodulatory properties of hASCs, limited ability to use hASCs from patients with obesity, IBD and/or cancer in an autologous ASC based therapy is recommended. That may be due to the repeatedly reported blunted immunosuppressive capacity of ASCs from those patients. In the context of other inflammatory and autoimmune diseases, such as RD, osteoporosis, and diabetes mellitus, contradictory results from being unaffected to slightly or severely affected ASC immunoregulatory capacity from patients with one of these diseases. To minimize discordant outcomes, the future studies need to take into account stratifying patients depending on patient-related parameters (such as age, body mass index, and genetic background) and disease-related parameters (such as the exact pathogenic cause, disease stage, duration). Importantly, the contradictory results can also be attributed to lack of standardized MSC immunopotency assays on both the whole PBMCs and a purified disease-relevant immune cell type [277]. Importantly, transciptomic and proteomic analyses of ASC/ immune cell cocultures with different incubation times will improve our understanding and knowledge for ASC immunomodulatory effects in vitro and factors mediating their actions over time in culture. Such recommended research would help to get data that can be systemically compared from different laboratories to draw solid conclusions.

ASCs may exert immunosuppressive potential in vitro; however, the real functionality should be ascertained in a disease model due to the significant differences b/w in vitro and in vivo conditions. Thus, there remains a need for complementary preclinical then clinical studies to identify different conditions affecting ASC immunomodulatory effects in vivo. In summary, the hASC immunomodulatory capacities in vitro widely vary depending on experimental setup, as well as donor-related factors. The above-mentioned microenvironemental determinants are recommended to be standardized by experts in the field to establish an effective hASCs immunosuppressive coculture setting.

### Supplementary Information

Below is the link to the electronic supplementary material.

Supplementary file1 (DOCX 63 KB)

## Data Availability

Not Applicable.
