# Supplementary material for: Factors Defining Human Adipose Stem/Stromal Cell Immunomodulation in Vitro
Source: Stem Cell Rev Rep. 2023 Nov 14;20(1):175–205. doi: 10.1007/s12015-023-10654-7 (PMC10799834; doi:10.1007/s12015-023-10654-7)
Supplement: Supplementary file 1 — Supplementary file1 (DOCX 63 KB) [file 12015_2023_10654_MOESM1_ESM.docx]

**Supplemental Table 1: Studies addressing the alloreactive and immunoregulatory effects of hASCs on T lymphocytes**

| Reference | ASC source | Immune cell type | Immune cell stimulant | Interaction type/  PBMC or T cell: ASC ratio | Assessed immune-related ASC parameters | Assessed immune cell-related parameters | Aim of the study |
| --- | --- | --- | --- | --- | --- | --- | --- |
| [51] | Abdominal Sc. AT | PBMCs | MLR, PHA, or surface-bound OKT3 | Direct (1:1)  Transwell/  Paracrine  (1:1) | -cytokines secretion | Proliferation | The study explored  - potential of hASCs to induce alloreactivity (immunogenicity)  -hASC vs hBMSC anti-proliferative effect on mitogen or alloantigen stimulated PBMC |
| [10] | Sc. lipoaspirate | T cells | MLR | Direct (40:1, 20:1, 10:1) | ---- | proliferation | The study aimed at addressing:  hASC immunogenicity  hASC Immunosuppressive potential of freshly isolated and early and late expanded ASCs |
| [60] | Lipoaspirate | PBMCs | MLR or PHA | Direct  Transwell  Different ratios up to 20:1 | ---- | -Proliferation  -IFN-ɤ, TNF-α,  and IL-12 secretion | The study aimed at addressing:  -hASC potential to induce alloreactivity  -hASC vs BMSC anti-proliferative effect on mitogen or alloantigen stimulated PBMCs  - the potential of ASC to control GVHD in mice transplanted with haploidentical hematopoietic Grafts |
| [11] | Lipoaspirate | PBMCs | MLR or PHA/IL-2 | Direct (1:1)  Paracrine (1:1) | -Paracrine mediators | Proliferation | The study aimed at addressing:  -hASC Immunogenicity  -hASC vs hBMSC anti-proliferative effect |
| [52] | Lipoaspirate | PBMC | MLR or PHA | Direct (1:1, 1:2, 1:4, 1:8)  Transwell (1:10) | ---- | Proliferation | The study aimed at addressing:  -hASC Alloreactivity  -hASC anti-proliferative effect on mitogen or alloantigen stimulated PBMC |
| [23] | Lipoaspirate | PBMCs,  CD4 and CD8 T cells | Microbeads loaded with anti-CD2, anti-CD3 and anti-CD28 | Direct (25:1)  Transwell (25:1) | -Surace expression of HLA-class I and II, CD40, CD80, CD86 | -Proliferation  -Proiflammatory cytokine production including IL-2, IL-5, IFN-ɤ, TNF-α) | To address the key role of IFN-ɤ and its induced molecule IDO in hASC immunosuppression |
| [88] | Lipoaspirate | PBMCs | MLR or PHA | Direct (1:1, 2:1, 4:1, 8:1 and 16:1) | -Ectopic expression of hTERT to establish human telomerase immortalized ASC | Proliferation | -hASC Alloreactivity  -hASC nti-proliferative effect on mitogen or alloantigen stimulated PBMCs |
| [81] | lipoaspirate | PBMCs | PHA | Direct (8:1) | -Cytokine production  -*IDO* expression | -Proliferation  -IFN, TNFα secretion | Comparative analysis of the immunomodulatory properties of hASCs vs BMSCs, CB-MSCs and  WJ-MSCs |
| [14] | Perirenal AT | PBMCs | No activation | Direct 5:1  Transwell 5:1 | -MSC expression of pro- and anti-inflammatory mediators and growth factors | -Proliferation,  -Activation markers (CD25&CD69)  -Functions as responder cells to mitogen and alloantigen stimulation and as immunosuppressive cells | This paper assessed mainly whether hASCs induce allogenic PBMC proliferation and the phenotype of the expanded T cells |
| [25] | Lipoaspirate | CD3 T cells | MLR or PHA/IL-2 | Direct (40:1 and 4:1) | -PGE2 and HGF secretion and Inhibition experiments | -Proliferation  -Activation markers expression (CD38) | To study the capacity of hASCs and WJ-MSCs to modulate lymphocyte reactions in response to different stimuli |
| [24] | Lipoaspirate | CD3 T cells | MLR or PHA/IL-2 | Direct (4:1, 8:1, 40:1 and 80:1) | -LIF expression at the gene and soluble protein levels | -Proliferation  -Treg expansion | This study aimed at exploring the capacity of MSCs isolated from AT and WJ, vs BM to modulate activated lymphocyte proliferation as well as their impact on regulatory T-cells. |
| [82] | ND | PBMCs | MLR or PHA | Direct (5:1)  Transwell (5:1)  Direct (1:1, 10:1, 100:1). | -PGE2 detection | -Proliferation  - Production of cytokines (IFN-ɤ, TNF-ɑ and IL-12, IL-10, IL-6 and IL-5)  -Gene expression of T cell subsets TFs | To compare the immunosuppressive effects of hASCs vs BMSCs on proliferation and cytokine production by activated PBMCs and the possible involvement of PGE2 in MSC immunomodulation |
| [61] | ND | PBMCs or  CD3 T lymphocytes | Surface bound αCD3 and soluble αCD28 for T cells and PBMC or MLR or PHA for PBMCs only | Transwell (1:1) | -Surface expression of immunological and immunoregulatory markers such as PD-L1, PD-L2, CD106 (VCAM-1), CD166 (ALCAM), CD54 (ICAM-1) | -Proliferation  -Activation (CD69, C25, HLA-DR)  -Pro- and anti-inflammatory cytokines production | To assess the immunesupressive effects of hASCs vs hAMSCs on PBMCs and T cells activated by different stimuli. It also aimed at exploring IFN-priming influence on hASC and hAMSC- anti-proliferative effect. |
| [31] | Sc. abdominal AT | CD3 T lymphocytes |  | Direct (1:1, 10:1, 1000:1) | ---- | -Proliferation  -Expression of *IFNG* and *IL-2* genes and soluble proteins | The mechanisms underlying hASC immunosuppressive mechanisms |
| [30] | Perirenal AT | CD25^-/dim^ effector cells | MLR | Direct (10:1) | ---- | -Frequency of Tregs  -Immunosuppressive functionality of Tregs generated in the presence of ASC  -Quantitative DNA methylation analysis of FOXP3-TSDR  -IL-2 expression analysis in the co-culture supernatant and the implication of this cytokine in ASC- mediated generation of Tregs | The study addressed whether hASCs induce *denovo* generation of Tregs or expansion of natural Tregs and the implication of IL-2 in such induction effect. |
| [70] | ND | PBMCs | PHA | Direct (10:1)  Transwell | ---- | -Viability  -Proliferation  -T cell activation (CD69, -CD25 and HLA-DR)  Th1/Th2 Cytokine profile (IL-6, IL-8, TNF-ɑ, IFN-ɤ, IL-10, IL-1β, IL-12p70, IL-2, IL-4, and IL-5) | This paper explored the contribution of direct contact and different O2 levels (standard 20% vs reduced 5%) on the immunosuppressive properties of hASCs. |
| [12] | Lipoaspirate | CD3 T cells | anti-CD3/CD28 | Direct (10:1) | -Expression of MHC molecules and costimulatoy molecules  -Expression of surface immunomodulators CD200, CD274, adhesion molecules ICAM-1, costimulatory VCAM-1  NKC-activating ligand CD112, CD155, MICA/B, ULBP-1, ULBP-2, and ULBP-3  -Gene expression analysis for major MSC immunomodulators including genes coding for IDO (INDO), Cox-2 (PTGS2), iNOS (NOS2), and TSG-6 (TNFAIP6)  -IDO activity  -Immunogenicity | Proliferation | The study aimed to assess the immunological properties of hASCs vs BMSCs expanded in clinical grade platelet lysate and the implication of proinflammatory priming to their immunosuppression |
| [6] | AT was dissected from nine cadaveric pancreata donated for islet transplantation | PBMCs | Anti-CD3/CD28 Dynabeads | Direct  Different ratios from 3:1 up to 100:1 | -Immunophenotype  -Differentiation  -Metabolic activity/proliferation by MTT assay  -Cytokines secretion including IL6,IL8, TGF-β  -Expression of *IDO and IL6* | -PBMC Proliferation  -Proinflammatory cytokines IFN-ɤ, TNF-ɑ, IL-12 secretion | The study aimed to compare the immunomodulatory functions of hASCs, relative to BMSCs, on lymphocytes and dendritic cells |
| [83] | Lipoaspirate | CD3 T cells | PHA/ IL2 | Direct (4:1) | -Cytokine secretion (IFN-ɤ, IL8, CCL5) after priming with a proinflammatory cytokines mix | -Proliferation  -Activation markers expression (CD23, CD26, CD45 and CD69)  -T cell migration  -Cytokines secretion | To assess the differences, b/w hASCs and WJ-MSCs vs BMSCs on proliferation, activation, and migration of activated CD3 T cells. The impact of inflammatory priming of MSCs on their immunosuppressive activity was also studied |
| [37] | ND | PBMCs | PHA | Direct (10:1) | ---- | -Activation stages of different lymphocytes including T cells in terms of expression of CD25, CD69 and HLA-DR  -Gene Expression of *IL-2, T-bet, GATA3* and *FOXP3* in T cells | This study aimed to compare the effects of PHA-activated PBMC coculture with hASCs vs BMSCs and UCM-MSCs on activation of lymphocytes including T cells |
| [63] | Lipoaspirate | PBMCs | PHA | Direct (2:1  10,: 1 100:1)  Transwell was mentioned but without details | -Characterization  -Proliferation | -Proliferation  -Cell cycle  -Apoptosis  -Activation CD69 (12hrs), CD25 (24hs),  CD44 (72hrs)  -Cytokines production (IFN-ɤ, IL2, IL10, IL4) | The study aimed to compare the immunomodulatory functions of hASCs vs hBMSCs on T cells *in vitro* |
| [84] | Lipoaspirate | PBMCs | PHA | Direct 8:1  Transwell 8:1 | -Expression of HLA-ABC, HLA-DR and PD-L1/B7-H1 (before and after IFN-ɤ treatment) | Proliferation | The study aimed to verify the role of B7-H1 and HLA-DR in the immunosuppressive effect of hASCs and other MSC populations |
| [90] | lipoaspirate | PBMCs, CD4 and CD8 T cell subsets | Anti-CD3,CD2,CD28 coated beads | Transwell (25:1) | ---- | -Proliferation  -Viability  -Immunomodulators measurement as PGE2, HGF and IL-10 in the co-culture supernatants | This study explored the relative contribution of each arm of the Trp/Kyn balance on the IDO-mediated immunomodulatory mechanisms of hASCs |
| [167] | Lipoaspirate | PBMCs | PHA | Direct (40:1) | -HLA class I and II mRNA expression  -Cytotoxicity by IL-2-treated PBMCs | Proliferation | This study addressed the temporal HLA expression profile and immunomodulatory function of hASCs vs BMSCs during *in vitro* expansion and whether HLA-G involved in the immunosuppressive effects of MSCs |
| [161] | Lipoaspirate | PBMCs | MLR or PHA | Direct  transwell  PBMC: MSC  (2:1, 10:1) | -HLA-Class I and II expression  -TGF-β and IL-2 expression  -IFN-ɤ priming  -Chondrogenic differentiation | -Proliferation  -T cell cycle  -T cell apoptosis  -T cell early activation marker (CD69 and CD25: 24 hours and CD44: 72 hours  -Cytokine production;  IFN-ɤ. IL-2, IL-4, IL-10 | This paper explored the involvement of Jagged-2 (Notch ligand 2) in the immunosuppressive effect of naïve, primed or chondrogenically differentiated hASCs, |
| [89] | Epicardial adult fat | T cells | Allogenic mature monocyte-derived dendritic cells | Direct (20:1; 40:1) | ---- | -Proliferation  -Viability  -Th1/Th2 cytokines secretion | This paper aimed to assess the immunomodulatory properties of epicardial hASCs relative to UCB-MSCs |
| [64] | ND | CD3 T cells | PHA | -Direct  (10:1; 20:1; 50:1) | ---- | -T cell proliferation  -Pro- and anti-inflammatory cytokines production | The study analyzed the in vitro immunomodulatory effects of hASCs vs BMSCs obtained from the same donor. |
| [95] | Lipoaspirate of Sc. abdominal fat | CD3 T cells | MLR or PHA | - Direct (1:1) | -IL-6 and IL-10 levels in the CM  -The expression of *IL-6R, TGF-β1, IP-10,* and *HGF-β* genes | -Proliferation  -Apoptosis  -Treg markers expression  -Analysis of cytokines gene or protein expression (IL-2, IL-6, IL-12, IFN-ɤ, TNF-ɑ, IL-17A and TGF-β1).  -Analysis for apoptosis-related genes expression | The study aimed to compare the immunosuppressive activities of hASCs vs BMSCs and WJ-MSCs |
| [32] | Skin fat | PBMCs | PHA or plate-bound anti-CD3 and anti-CD28 antibodies | Direct (25:1)  Transwell (25:1) | -Surface expression of CD54/ICAM-1  -IDO and iNOS expression | -Proliferation  -CD25 expression  -CD54 expression | The authors studied the molecular mechanisms underlying hASC immunomodulation and the role of ICAM-1 in direct and IDO in transwell contact |
| [33] | Sc. AT | PBMC, CD4 and CD8 T cell subsets | anti-CD3 and anti-CD28 antibody-coated beads | Direct (5:1)  Transwell (5:1) | -Surface Expression of  PD-L1 and Gal-9 | -Cell proliferation  -apoptosis  -Intracellular IFN-ɤ production  -Surface Expression of PD-1 and TIM-3 - NF-κB activation | To investigate the mechanisms underlying the inhibitory effect of hASCs on T cells. |
| [65] | Subcutaneous AT and intra-abdominal fat of omentum | PBMCs | MLR or PHA | Direct  Transwell  (2:1, 4:1, 8:1, 16:1) | -Surface phenotype  -Analysis of cytokines production | -Proliferation  -Cytokines production | This study aimed to investigate the immunomodulatory properties of Sc. And o.hASCs vs BMSCs derived from the same individual to validate their applicability for transplantation across MHC barriers |
| [99] | Lipoaspirate | PBMCs | PHA | Direct  Paracrine | -Viability  -Immunophenotype  -ASC growth and Migration  -ROS levels | Proliferation  -Cytokines production | The study aimed to describe the reciprocal effects of allogeneic hASCs and activated PBMCs. |
| [93] | Lipoaspirate | CD3 T cells | PHA/IL-2 | Low cell ratio (5:1)  High Cell ratio (80:1) | -ASC priming with a proinflammatory cytokines mix | -Flow cytometer analysis to detect the percentage of IL-17 producing cells  -Analysis of cytokines involved in Th17 pathway | The study aimed to demonstrate Th17 immune response modulation in the presence of hASCs with focus on the impact of cell ratio and hASC priming |
| [17] | AT of abdomen or breast | Mouse CD3 T cells | Plate coated anti-CD3/soluble anti-CD28 | Direct (1:.7) | -The surface expression of human leukocyte antigens; HLA-ABC, HLA-DR, costimulatory molecules; CD80 and CD86 and NKG2D ligand; MIC-A, MIC-B, ULBP-1 and ULBP-2/5/6 | -Proliferation of stimulated mouse CD3 T cells  -generation of alloreactive and memory CD8 T cells during allogenic stimulation | The study addressed the immunogenicity of allogeneic human hASCs through the production of alloreactive-CD8 T and -memory CD8 T cells |
| [22] | ND | PBMCs or CD4 T cells | PHA/IL-2 | Direct or Transwell | Intracellular measurement of IDO in hASC after treatment with IFN-ɤ | -Proliferation | The study assessed the immunomodulatory strength, on T cells, of different human MSC sources including AT, relative to  IFN-ɤ primed MSC CM and EV and the molecular mechanisms involved in MSC immunosuppression |
| [58] | Abdominal subcutaneous AT | CD3 T cells | PHA | Direct 10:1 | --- | Morphology  Viability  Proliferation  Activation Markers expression  % of T cell subsets  Gene expression for *IL2, CCL3, TGF-B1, PDCD1, IL10, IFNG, Foxp3*  Th1/Th2 11 cytokines analysis by flow cytometry and cytokines secretion by ELISA | The authors studied the effect of hypoxia on the hASC immunosuppression on PHA- CD3+T cells was demonstrated. |
| [21] | Lipoaspirate | PBMCs | Anti-CD3/CD28  In some experiments, PBMC left unstimulated | Direct (5:1)  Transwell (5:1) | -IDO expression and activity | -Proliferation  -Treg markers expression  -pro- and anti-inflammatory cytokines secretion | -The study analyzed the immunomodulatory potential of hASCs, on CD4 T cells, addressing potential cell-contact dependency in relation to T cell receptor stimulation of whole PBMCs  -The study also assessed frequency of Tregs in co-culture with stimulated or non-stimulated PBMCs |
| 110] | ND | PBMCs | T cell activation/ expansion kit (beads with antibodies against CD2,CD3 and CD28) | Transwell (4:1) | -Expression of soluble immunomediators such as IDO, IL1RA, and PGE2 and surface immunomodulators including ICAM-1, VCAM-1, and PD-L1 in response to priming by pro-inflammatory cytokines IFN-ɤ and TNF-ɑ | -Proliferation  -CD8, CD25 and CD69 expression levels | The study examined the immunoregulatory functionality of both female and male hASCs in order to gain further insights into donor selection. |
| [87] | Lipoaspirate | PBMCs | PHA | Direct (4:1 or 40:1) | --- | - proliferation was measured by the percentage of CD38 positive cells in the whole population  - IFN-ɤ and TNF-ɑ cytokines measurement | The study aimed to evaluate the immune modulation of SVF and MSCs from the same SVF samples to support an explanation of when SVF or MSCs should be used |
| [67] | Sc. AT from abdomen or breast | CD4 T cells | Plate bound anti-CD3/CD28 | Direct (5:1) | -Expression of PD-L1, CD54, MHC class II and II and the costimulatory molecules (CD40, CD86) before and after priming  -Expression of IDO in response to IFN-ɤ treatment | -Proliferation  -Activation markers analysis  -Cytokines release  -Tregs and intracellular cytokines expression | The study aimed to evaluate the impact of T2D and obesity on the immunosuppressive functions of hASCs on CD4 T cells |
| [59] | Liposuction from different anatomical areas such as the abdomen, breast and hip | γδ T cells in heterologous experiments and PBMCs in autologous settings |  | Direct (5:1 and 10:1)  or CM from spheroid or adherent ASCs | -the expression of 51 secreted proteins in ASC secretome  (spheroid vs adherent ASC cultures) | -proliferation  -Intracellular cytokines expression | The study aimed at analyzing the immunomodulatory properties of hASC spheroids, relative to the traditional ASC monolayer |

AT: Adipose tissue, ASCa: adipose tissue-derived mesenchymal stem/stromal cells, ALCAM: Activated leukocyte cell adhesion molecule, BMSCs: bone marrow-MSCs, CD: Cluster of Differentiation, CM: Conditioned medium, COX/PTGS: Cyclooxygenase/ Prostaglandin-endoperoxide synthase, CB: Cord Blood, ER: Endoplasmic reticulum, EV: Extracellular vesicle, FOXP3, Forkhead box P3, GVHD: Graft versus host disease, Gal: Galectin, HGF: Hepatocyte growth factor, HLA: Human Lekocyte antigen, hAMSCs: human amniotic membrane-derived MSCs, hASCs: human ASCs, IL: Interleukin, IDO: Indoleamine 2,3 dioxygenase, iNOS: Inducible nitric oxide synthase, IFN-ɤ: Interferon-gamma, ICAM: Intracellular cell adhesion molecule, ILF: Leukemia inhibitory factor, MLR: Mixed lymphocyte reaction, MICA/B: MHC class I chain related-proteins A and B, NK: Natural Killer, NKG2D, Natural Killer Group 2D, [NF-κB: nuclear factor kappa-light-chain-enhancer of activated B cells, O: Omentum/omental, PD/ PDCD: Programmed death/ Programmed death cell death, PD-L: Programmed death ligand, PGE2: Prostaglandin E2, PBMCs: peripheral blood mononuclear cells, PHA: phytohaemagglutinin, ROS: Reactive oxygen species, SVF: Stromal vascular fraction, Sc: Subcutaneous, T2D: Type 2 diabetes, TNF-ɑ: Tumour necrosis factor alpha, hTERT, Human telomerase reverse transcriptase, TNFAIP6: TNF-ɑ induced protein 6, TGF-β: Transforming growth factor beta, TIM-3: T cell immunoglobulin and mucin-domain containing-3, TSG-6: Tumor necrosis factor-stimulated gene 6, Trp/Kyn: Tryptophan/Kynurenine, Th: T helper cells, TSDR: Treg-specific de-methylated region, TF: Transcription factor, UCM: Umbilical cord matrix, UCB: Umbilical cord blood, ULBP: UL16 binding protein, VCAM: Vascular cell adhesion molecule, WJ: Wharton’s Jelly.](https://en.wikipedia.org/wiki/NF-%CE%BAB)
